# Extinction-restraint intervention eradicates fear memory encoded in BLA engram cells via D2 receptor-mediated dopaminergic modulation from locus coeruleus

**DOI:** 10.1038/s12276-026-01794-0

**Published:** 2026-08-13

**Authors:** Tongxia Li, Jian Yang, Chi Cui, Kun Ren, Lijun Zhang, Ming Li, Xueke Yang, Xiang Peng, Jie Lei, Yulong Shi, Gangan Luo, Yibo Yao, Junsong Du, Sitong Chen, Jie Ming, Xuemin Wang, Pei Zhang, Bo Tian

**Affiliations:** 1 Hubei Provincial Clinical Research Center for Alzheimer’s Disease, Tianyou Hospital, School of Medicine, Wuhan University of Science and Technology, Wuhan, Hubei China; 2 Department of Neurology, Tongji Hospital, Tongji Medical College, Huazhong University of Science and Technology, Wuhan, Hubei China; 3 Department of Neurobiology, School of Basic Medicine, Tongji Medical College, Huazhong University of Science and Technology, Wuhan, Hubei Provin China; 4 Department of Breast and Thyroid Surgery, Union Hospital, Huazhong University of Science and Technology, Wuhan, Hubei Province China; 5 Department of Neurobiology, Southern Medical University, Guangzhou, Guangdong Province China; 6 Hubei Province Key Laboratory of Occupational Hazard Identification and Control, Wuhan University of Science and Technology, Wuhan, Hubei Province China; 7 Academy of Advanced Interdisciplinary Research, Wuhan University of Science and Technology, Wuhan, Hubei Province China

**Keywords:** Post-traumatic stress disorder, Diseases of the nervous system

## Abstract

Despite undergoing exposure therapy, individuals with post-traumatic stress disorder (PTSD) frequently experience relapses when confronted with trauma reminders. This study examined the impact of extinction-restraint intervention (Ext-Res) in a PTSD-related fear relapse model. We found that reactivation of basolateral amygdala engram cells (BLA^engram^) correlates with relapse following fear reinstatement. Post-extinction BLA^engram^ activation induced fear relapse, whereas Ext-Res effectively protected relapse and facilitated BLA^engram^ reactivation. Our investigation into the neural circuitry connecting the tyrosine hydroxylase (TH)-positive locus coeruleus neurons (LC^TH^) and BLA^engram^ revealed that dopamine release, but not norepinephrine, is crucial for the deactivation of BLA^engram^ following Ext-Res. Manipulation of the LC-BLA circuit could either replicate or reverse the therapeutic effects of Ext-Res. Notably, dopamine D2 receptors emerged as vital mediators of these effects. Overall, we reveal that Ext-Res intervention disrupts fear memory encoded in BLA^engram^ through dopamine D2 receptor-dependent manner, providing new insights into potential therapeutic strategies for preventing relapse in PTSD.

## Dopamine D2 receptors block fear memory reactivation in PTSD

Post-traumatic stress disorder often persists despite exposure therapy, as patients may relapse when faced with traumatic triggers. This study explores the mechanisms behind fear relapse and potential interventions using a mouse model. Researchers identified that fear reinstatement reactivates memory engram cells in the basolateral amygdala (BLA), which are crucial for fear memory. They used optogenetic and chemogenetic tools to manipulate these cells and circuits, revealing that extinction-restraint intervention can erase fear memory traces. This intervention activates the locus coeruleus neurons, which project to BLA engram cells, releasing dopamine that acts on D2 receptors to prevent fear relapse. These findings suggest that targeting the locus coeruleus-BLA circuit and dopamine signaling could enhance post-traumatic stress disorder treatments, offering a new therapeutic avenue to prevent relapse after exposure therapy. This summary was initially drafted using artificial intelligence, then revised and fact-checked by the author.

## Introduction

Post-traumatic stress disorder (PTSD) is a psychiatric disorder that often develops soon after exposure to one or more unusual threats or horrific events^1,2^. Clinically, exposure therapy is one of the effective clinical treatments for treating PTSD, and it has also been verified to relief clinical symptoms^3^. However, even after exposure therapy, patients with PTSD may still face traumatic triggers, which can lead to repeated relapses^4^. In experimental animals, fear conditioning paradigms have been widely used as a classical model of PTSD^5^. Fear conditioning generates fear memory, which can be extinguished by repeated exposure to conditioned stimulus (CS) in a safety environment such as exposure therapy. Fear reinstatement (FR) can trigger relapse whereby extinguished fear with the CS (for example, pure tones) returns following re-exposure to the unconditioned stimulus (US, for example, footshock) alone^6^. The mechanism underlying FR-evoked relapse of extinguished fear involves memory engram cells^7^. Recent studies have suggested that engram cells established from fear conditioning were identified in several brain areas, especially in basolateral amygdala (BLA)^8,9^. Activation of BLA engram cells (BLA^engram^) by optogenetic tool is sufficient to drive freezing behavior^9^, indicating that fear memory may be encoded and stored in BLA^engram^. Moreover, FR is able to reactivate BLA^engram^ labeled during fear conditioning^6^. Thus, it is important to clarify the mechanism underlying how the BLA^engram^ contributes to relapse of extinguished fear, which also can provide new insight into the pathophysiological mechanism of PTSD relapse after exposure therapy.

Fear extinction following a repeated exposure to the CS enables fear memory into a labile state called reconsolidation, which requires the reactivation of engram cells and provides the opportunity to modify or erase an undesired fear memory^7^. Although both memory reconsolidation and extinction involve changes to fear memory, reconsolidation refers to the process of updating or modifying an existing fear memory after its reactivation, whereas extinction involves reducing the fear response to a previously learned stimulus through repeated exposure without reinforcement. Also, the time window of reconsolidation normally lasts from 10 min to 6 h (ref. ^10^). In a human study, a fear memory trace can be erased by disrupting reconsolidation with extinction training through an amygdala-dependent mechanism^11^. Infusion of the protein synthesis inhibitor anisomycin into the BLA immediately after a single CS presentation-induced fear memory reactivation impairs future fear retrieval^12^. Meanwhile, BLA neurons receive direct inputs from the brainstem locus coeruleus (LC), and activation of these projections is sufficient to produce extinction deficits^13^, which reflect that LC-BLA circuit may involve in fear memory reconsolidation. Activation of LC tyrosine hydroxylase (TH)-positive (LC^TH^) neurons drives the corelease of norepinephrine and dopamine into the downstream brain regions, such as BLA and hippocampus^14,15^. Rodent behavioral studies using pharmacological manipulation of BLA noradrenergic receptors and dopamine receptors also provide much of the evidence, with the source of these neurotransmitters thought to be the LC^TH^ neurons^16–19^.

Previously published studies have been suggested that acute restraint stress enhances LC^TH^ neurons and augments norepinephrine and dopamine release^20–22^. The beneficial effects of acute restraint stress were observed in learning and memory via synaptic transmission and plasticity^23,24^. Moreover, acute restraint stress is able to obviously interrupt the memory consolidation process after memory acquisition, and certain types of stress can successfully disrupt memory reconsolidation^25,26^. We thus reasoned that restraint following extinction (extinction-restraint, Ext-Res) intervention may have a positive impact on fear relapse. In addition, pharmacological blockade of dopamine D1 receptors in infralimbic cortex protects against FR-triggered relapse of extinguished fear^27^, indicating that dopamine and its receptors probably as a potential therapeutic target for preventing PTSD relapse. However, whether the LC^TH^ neurons send projections to BLA^engram^ and how these projections and dopamine signaling participate in the treatment of relapse of extinguished feat by Ext-Res intervention remain incompletely understood.

Here, we identified an increase of reactivated BLA^engram^ specifically in FR-induced relapse of extinguished fear mouse model by using activity-dependent cell tagging (activity-tagging) technique. Artificially activation of BLA^engram^ directly results in increased fear response. Ext-Res intervention efficiently erased the fear memory trace encoded by BLA^engram^, which is driven by input from the LC^TH^ neurons. We also demonstrated that LC^TH^-BLA circuit is not only necessary but also sufficient for the therapeutic effect of Ext-Res intervention on relapse of extinguished fear. Thus, this study proposes that an increased reactivity of BLA^engram^ may be crucial for relapse of extinguished fear and uncovers a previously undescribed circuit mechanism for erasing fear memory trace by Ext-Res intervention.

## Materials and methods

### Animals

All animal experiments were conducted in compliance with the NIH Guide for the Care and Use of Laboratory Animals. The experiments received approval from the Animal Care and Use Committee of Huazhong University of Science and Technology, Wuhan, Hubei, China. Wild-type (WT) C57BL/6J mice, aged 8~10 weeks, were procured from Beijing Vital River Laboratory Animal Technology, Beijing, China. TH-Cre transgenic mice (JAX stock no. 008601) were sourced from Jackson Lab, CA, Bar Harbor, Maine, USA^28^. The experiments utilized adult mice aged 8~10 weeks. Before the experiments, the mice were group-housed in standard laboratory cages (3~5 mice per cage). The ambient temperature was maintained at 22 ± 2 °C with a 12-h light–dark cycle (lights on from 08:00 to 20:00). The mice had ad libitum free access to water and food.

### Viral vectors

The viruses, including AAV9-c-Fos-tTA, AAV9-TRE-Cre, AAV9-TRE-ChR2-mCherry, AAV9-TRE-mCherry, AAV9-DIO-hM3Dq-mCherry, AAV9-DIO-hM4Di-mCherry, AAV9-DIO-mCherry, AAV9-DIO-EGFP, AAV9-DIO-TVA-EGFP, AAV9-DIO-G, RV-EnVA-ΔG-dsRed, AAV9-DIO-GCaMP6m, AAVretro-TH-Cre, AAV9-DIO-NE2h, and AAV9-DIO-DA3m were purchased from BrainVTA Co., Ltd (Wuhan, China). The concentrations of viruses were used for stereotaxic injection range of 3~8 × 10^12^ genome copies per milliliter. Viral vectors were subdivided into aliquots stored at −80 °C until use.

### Stereotaxic surgery

For stereotaxic injection of viruses, mice were first anesthetized using 2% isoflurane. Following this, mice were carefully positioned on a stereotactic instrument (68030, RWD Life Technology Co. Ltd, China). To ensure the well-being of the mice after the procedure, a heating pad was utilized to maintain their body temperature at a steady 37 °C. Microinjections were carried out using a microinjection pump (53311, Stoelting, USA). The glass capillaries (504949, WPI, USA) with a tip diameter ranging from 10 μm to 20 μm were connected to a 10 μl microsyringe (Hamilton, NV, USA) to facilitate the injection process. The speed of virus injection was set at a rate of 20 nl/min. After each injection, the syringe was left for 10 min to allow the spread of virus. The injection coordinates of each brain region were displayed as follows: anterior–posterior (AP): −1.00 mm, mediolateral (ML) from bregma: ±3.10 mm, dorsal–ventral (DV) from the dura: −4.95 mm for BLA; AP: −5.20 mm, ML: ±0.75 mm, DV: −4.20 mm for LC.

For engram cell labeling and fiber-photometry experiments, mice were unilaterally injected with AAV9-c-Fos-tTA and AAV9-TRE-mCherry (1:1, 200 nl), AAV9-c-Fos-tTA, AAV9-TRE-Cre, and AAV9-DIO-GCaMP6m (1:1:1, 200 nl) into the BLA. For optogenetic activation, mice were bilaterally injected with AAV9-c-Fos-tTA and AAV9-TRE-ChR2-mCherry (1:1, 200 nl) into the BLA. For chemogenetic manipulations, mice were bilaterally injected with AAV2/retro-TH-Cre (200 nl) into the BLA and AAV9-DIO-hM3Dq-mCherry, AAV9-DIO-hM4Di-mCherry, or AAV2/9-DIO-mCherry (200 nl) into the LC. For the anterograde tracing of the circuit from LC^TH^ neurons to BLA engram cells, TH-Cre transgenic mice were unilaterally delivered AAV9-DIO-EGFP into the LC (200 nl), and a mixture of AAV9-c-Fos-tTA and AAV9-TRE-mCherry (1:1, 200 nl) into the BLA. The optical fibers (1.25 mm, Ø = 200 μm, NA = 0.37, Inper Technology Co., Ltd) were implanted 0.1~0.3 mm above the injection site immediately following the viral injection.

### Behavioral paradigms

#### Fear conditioning

For fear conditioning on day 1, mice were in a square chamber with length 30 cm, width 30 cm, and height 50 cm (context A, 30 cm × 30 cm × 50 cm). Each chamber contained an electrifiable metal-grid floor and a sound speaker. The mice were explored freely for 120 s without any stimulation and then subjected to three pairings of an auditory tone (CS, 30 s, 11 kHz, 75 dB) that co-terminated with a 2 s, 0.65 mA footshock of US. The interstimulus interval (ITI) between each pairing was set as 60 s. For fear recall on day 2, mice were in a perceptually different context which is a rounded chamber with 32 cm diameter and 50 cm height (context B). The electrifiable metal-grid floor was replaced by a white plastic floor. After 120 s acclimatization, three 30 s auditory presentations with 60 s ITI were administered. For extinction during days 3~5, mice received 12 trails of 30 s CS with 60 s ITI in context A every day. Data for this session were binned into four bins throughout the 12 trails of CS. For FR on day 6, FR mice were exposed to three consecutive footshocks (0.65 mA, 2 s) and with an 88 s ITI in context A, whereas the non-FR (NFR) group mice were placed in context B without footshock stimulus. For fear retrieval on day 7, all mice were subjected to three trails of CS in context B to mimic the relapse of extinguished fear. For Ext-Res intervention during days 3~5, mice were restrained immediately following extinction for 6 h every day in a 50 ml cylindrical plastic tube with small round holes in pipe wall, which allows them to breathe normally.

### Open field test

The open field test was performed to assess general locomotor activity and anxiety-like behavior. Mice were individually placed in the center of a square open-field arena (50 × 50 × 50 cm) and allowed to explore freely for 5 min. Locomotor activity was recorded and analyzed using SuperMaze software (Shanghai Xinruan Information Technology, China). The central zone was defined as a square region (30 × 30) in the center of the platform. Time spent in the center zone and distance traveled within the center were quantified for each animal.

### Elevated plus maze test

The elevated plus maze consisted of two open arms (35 cm × 5 cm), two closed arms (35 cm × 5 cm, with 10-cm-high walls), and a central open area (5 cm × 5 cm). The maze was elevated 75 cm above the floor. Each mouse was gently placed in the central area facing an open arm and allowed to explore freely for 5 min. Behavior was recorded and analyzed using SuperMaze software. The time spent in the open and closed arms and the number of entries into each arm were quantified.

### Grip strength test

The grip strength test was used to assess forelimb and hindlimb muscle strength and general motor function. The grip strength meter was positioned horizontally and equipped with a metal grid connected to the force transducer. Each mouse was gently placed onto the metal grid and allowed to grasp it with all four limbs. The mouse was then steadily pulled backward by the tail in a horizontal direction until it released the grid. The peak force value was automatically recorded by the device. Each mouse was tested three times with brief intervals between trials, and the average of the three measurements was used for subsequent analysis.

### Rotarod test

Motor coordination and balance were evaluated using the rotarod test. The rotarod apparatus consisted of a rotating rod with a diameter of 6 cm. Mice were habituated to the testing room for at least 2 h before testing. Before the formal test, mice underwent an adaptation training session in which the rod rotated at a low speed (5–10 rpm) for 10 min to allow the animals to learn to walk on the rotating rod.

### Novel object recognition test

The novel object recognition test was conducted in a square white arena (50 × 50 × 50 cm) to evaluate recognition memory and exploratory behavior. On the training day, two distinct objects — a cylindrical object (object A) and a cubic object (object B) — were placed symmetrically in the arena. Mice were allowed to freely explore the arena for 5 min. On the test day (24 h after), object B was replaced with a novel object (object C; triangular pyramid). Objects differed in shape and color but were made of the same material (wood) and placed in the same locations as during training. Mice were allowed to explore for an additional 10 min. Behavioral activity was recorded and analyzed using SuperMaze software. Exploration was defined as the mouse directing its nose toward an object less than 2 cm. Total exploration time was quantified, and the di index for each object was calculated as the proportion of time spent exploring that object relative to total object exploration time.

### Fiber photometry recording and analysis

The excitation wavelength of 470 nm was used for the fluorescence emission of calcium indicator GCaMP6m or the neurotransmitter sensors. The excitation wavelength of 410 nm served as the internal control. Both wavelengths operated at a frequency of 15 Hz with an exposure time of 30 ms. The optical fiber was connected to the fiber photometry system and aligned to record the fluorescence signals during the CS stimulus of fear retrieval, blue light stimulation, clozapine-*N*-oxide (CNO) injection, or restraint treatment. For neurotransmitter release recordings, gene-encoded norepinephrine sensors (NE2h) and dopamine sensors (DA3m) were used. Previously published studies indicated that NE2h exhibits an EC₅₀ for norepinephrine of ~190 nM, with rapid on-kinetics (*τ*_on_: 0.085 s) and off-kinetics (*τ*_off_: 1.6 s) under fast ligand application conditions in vitro. The DA3m sensor displays an apparent EC₅₀ for dopamine of ~90 nM, with similarly fast on-kinetics (*τ*_on_: 0.054 s) and sub-second off-kinetics (*τ*_off_: 0.57 s). Importantly, at physiologically relevant concentrations, NE2h and DA3m exhibit high selectivity for their respective ligands^29,30^. In these experiments, Δ*F* is defined as the difference between the recorded fluorescence transient and the basal *F*_0_ value, which is defined as the median fluorescence transients before the onset of salient events. All recordings were initiated after the photometry signal stabilized. The GCaMP6m signals were quantified by normalizing the changes of their fluorescence signals (Δ*F*/*F*_0_). The neurotransmitter sensor signals were standardized using *Z*-scores with a baseline of the 2 min preceding binding. Analysis was conducted in 2-min intervals within the 14 min preceding recording to characterize the temporal dynamics of neurotransmitter release. The data were analyzed with a custom-written MATLAB code, which has been uploaded in public database (https://github.com/BehavioralLAB/Fiberphotometry).

### Optogenetic and chemogenetic manipulation

**Fig. 1:**
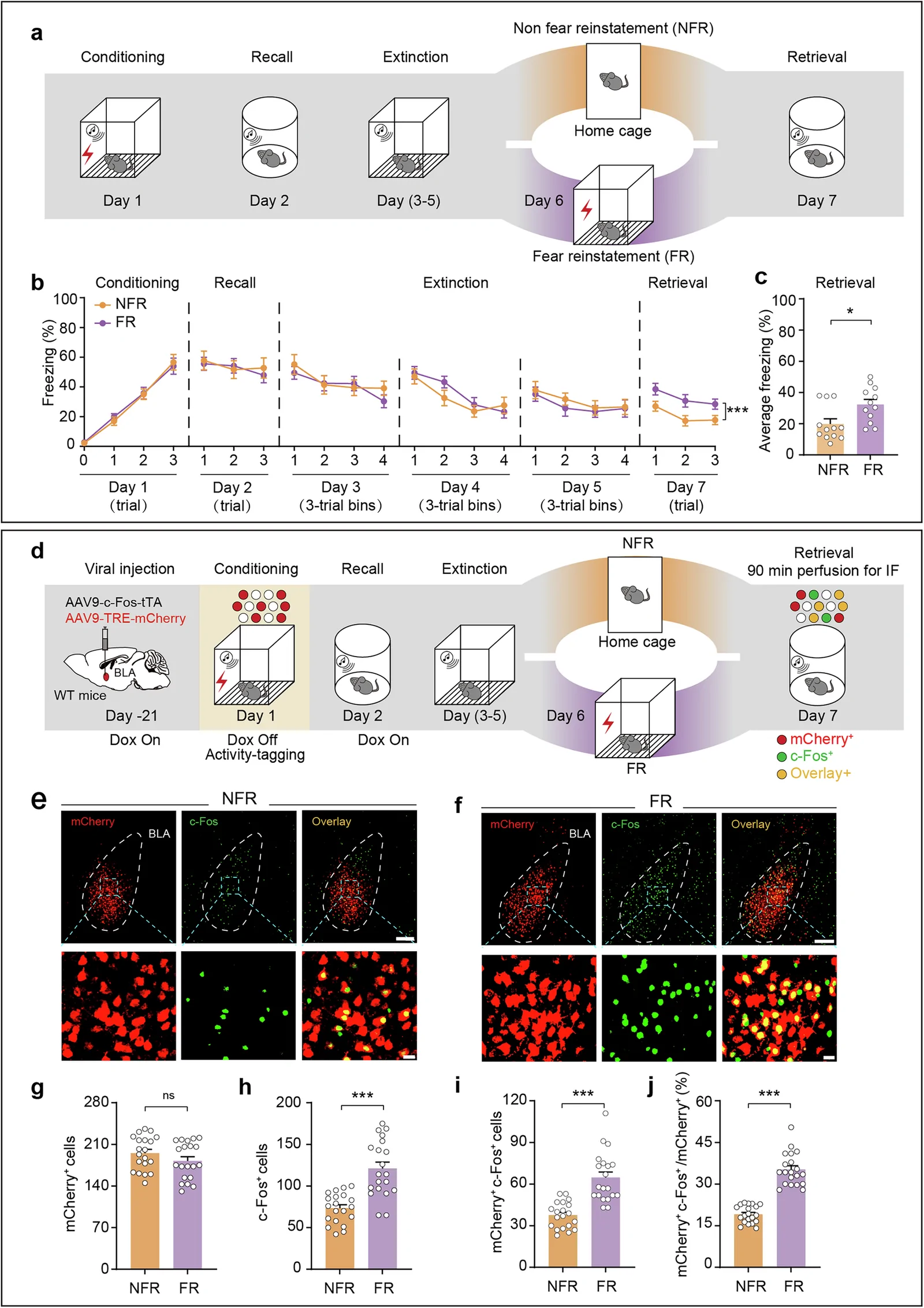
FR after extinction causes fear relapse and reactivates BLA engram cells. **a** Schematic diagram of the experimental design. **b** Freezing levels of conditioning on day 1, recall on day 2, extinction during days 3~5, and retrieval on day 7 (retrieval phase: NFR versus FR: *F*_(1,66)_ = 16.25, *P* = 0.0001; *n* = 12 mice per group). **c** Average levels of freezing response in retrieval phase (NFR versus FR, *t* = 2.684, *P* = 0.0135). **d** Dual labeling strategy of BLA engram cells. Cells activated by fear conditioning (day 1) were selectively expressed with mCherry (red), and the cells activated by the CS (day 7) in fear retrieval session were stained with the c-Fos primary antibody (green). Representative images of BLA mCherry^+^ (red) and c-Fos^+^ (green) cells in NFR (part **e**) and FR (part **f**) mice (top: scale bar, 500 µm; bottom: scale bar, 25 µm). Results of cell counts for mCherry^+^ cells (part **g**: *t* = 1.378, *P* = 0.1762), c-Fos^+^ cells (part **h**: *t* = 5.531, *P* < 0.0001), and mCherry^+^c-Fos^+^ overlap cells (part **i**: *t* = 5.960, *P* < 0.0001). **j** Percentage of mCherry^+^c-Fos^+^ cells in mCherry^+^ cells (*t* = 10.84, *P* < 0.0001). For parts **g**–**j** there are 20 slices from 5 mice for each group. Significant differences were determined by two-way analysis of variance with post hoc Sidak multiple comparisons (part **b**), and two-tailed unpaired *t* test (parts **c**and **g**–**j**). Data are presented as mean ± SEM. *P < 0.05, **P < 0.01, ***P < 0.001, and ns for no significance. BLA, basolateral amygdala; IF, immunofluorescence; WT, wild-type.

For optogenetic manipulation of BLA engram cells, the activated BLA engram cells were labeled by ChR2-mCherry. The output of 473 nm blue light was measured by an optical power meter and adjusted to 4~6 mW (20 Hz, pulse width 10 ms). After freely exploring for 120 s in a new context, mice received 30 s optical stimulation with 30 s interval for three times. The freezing behavior of mice in each session (three repeats of light off and light on) was recorded and analyzed by SuperMaze software (XinRuan Technology Co. Ltd). Similarly, the changes of calcium fluorescence signal in BLA engram cells were recorded following the activation of LC^TH^ neurons by using these parameters of optogenetic stimulation (Fig. 1l–q).

For chemogenetic manipulation of the projections from LC^TH^ neurons to BLA, mice were intraperitoneally injected with CNO (C0832, Sigma; 1 mg/kg, i.p.) or equal volume saline to activate or silence these projections during the time window of memory reconsolidation.

### Slice electrophysiology recording

Mice were anesthetized with isoflurane and then decapitated to prepare acute brain slices. The brains were swiftly extracted and placed in a cutting solution that was oxygenated (95% O_2_ and 5% CO_2_) and chilled to ice cold, containing 120 mM choline chloride, 2.5 mM KCl, 7 mM MgCl_2_, 0.5 mM CaCl_2,_ 1.25 mM NaH_2_PO_4_, 3.1 mM sodium pyruvate, 3.3 mM sodium L-ascorbate, 26 mM NaHCO_3_, and 10 mM glucose. Coronal sections were prepared using a vibratome (VT 1200S, Leica, Germany), resulting in slices with a thickness of 300 μm. Then slices were incubated at 32 °C in oxygen-saturated artificial cerebrospinal fluid (ACSF), comprising 124 mM NaCl, 3 mM KCl, 26 mM NaHCO_3_, 1.2 mM MgCl_2_, 1.25 mM NaH_2_PO_4_, 10 mM glucose, and 2 mM CaCl_2_ at pH 7.4 for 30 min. Following this initial incubation period, the slices were maintained at room temperature in oxygenated ACSF for an additional hour before being transferred to the recording chamber, which was set to 25 °C. During recording, ACSF was continuously perfused at a flow rate of 2 ml/min. Whole-cell recordings were performed under visual guidance. Patch pipettes were pulled from borosilicate glass capillaries (Warner Instruments, USA) using a P-1000 puller (Sutter Instruments, USA). Micropipettes had resistances of ~4–6 MΩ and were filled with an internal solution containing: 135 mM K-gluconate, 10 mM HEPES, 0.2 mM EGTA, 2 mM Mg-ATP, and 0.3 mM Na-GTP at pH 7.4. In current-clamp mode, CNO (10 μM) and sulpiride (10 μM) were applied via the perfusing ACSF to assess their effects on action potential firing in the patched neurons. Offline data analysis was performed using Clampfit 10.6 software (Molecular Devices).

### Intracerebral drug administration

For in vivo pharmacological manipulation experiments, we utilized a polyethylene tube to establish a connection between a 1 μl Hamilton syringe and a guiding cannula microinjector. This setup was used for intracerebral injections. All drugs were administered in 100 nl saline directly into the BLA. Following the injection, we left the inner tube of the cannula in place at the injection sites for over 3 min to facilitate drug diffusion. Three dopamine drugs were used in this study, including dopamine (0.5 μg in 100 nl saline, MedChemExpress), dopamine D1 receptor antagonist (SCH23390, 1 μg in 100 nl saline, MedChemExpress), and dopamine D2 receptor antagonist (sulpiride, 0.5 μg in 100 nl saline, MedChemExpress). The doses of dopamine and the D1R/D2R antagonists were selected based on well-established protocols from previous study^31^.

### Immunofluorescence staining and imaging

**Fig. 2:**
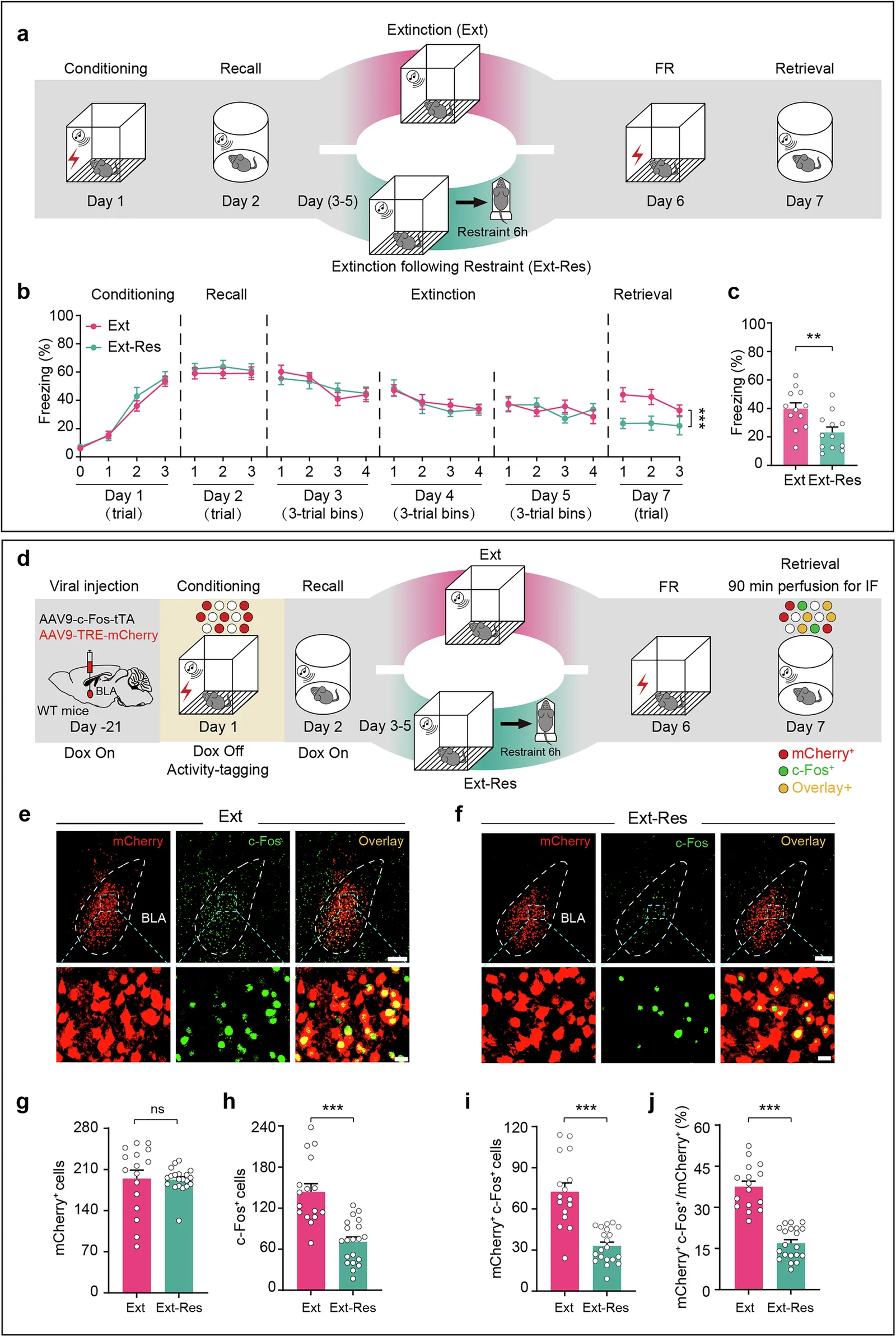
Ext-Res intervention ameliorates fear relapse and deactivates BLA engram cells. **a** Experimental timeline of fear conditioning, interventions, and behavior tests. **b** Mouse freezing responses of conditioning on day 1, recall on day 2, extinction during days 3~5, and retrieval on day 7 (retrieval phase: Ext versus Ext-Res, *F*_(1,66)_ = 16.91, *P* = 0.0001; *n* = 12 mice per group). **c** Statistical analysis of freezing levels during the retrieval phase (Ext versus Ext-Res, *t* = 2.987, *P* = 0.0068). **d** Schematic of the timeline for basolateral amygdala (BLA) engram cells activity-tagging in Ext and Ext-Res mice. Representative fluorescence images of BLA mCherry^+^ (red) and c-Fos^+^ (green) cells in Ext (part **e**) and Ext-Res (part **f**) mice (top: scale bar, 500 µm; bottom: scale bar, 25 µm). **g**–**i** Quantitative analysis of mCherry^+^ cells (part **g**: *t* = 0.1530, *P* = 0.8793), c-Fos^+^ cells (part **h**: *t* = 6.088, *P* < 0.0001), and mCherry^+^c-Fos^+^ overlap cells (part **i**: *t* = 8.871, *P* < 0.0001). **j** Proportion of dual labeling mCherry^+^c-Fos^+^ cells in mCherry^+^ cells (*t* = 8.871, *P* < 0.0001). For parts **g**–**j** there are 16 slices from 4 mice in the Ext group and 20 slices from 5 mice for the Ext-Res group. Significant differences were assessed by two-way analysis of variance with post hoc Sidak multiple comparisons (parts **b**and **c**) and two-tailed unpaired *t* test (parts **g**–**j**). Data are displayed as mean ± SEM. *P < 0.05, **P < 0.01, ***P < 0.001, and ns for no significance. Dox, doxycycline; FR, fear reinstatement; IF, immunofluorescence.

**Fig. 3:**
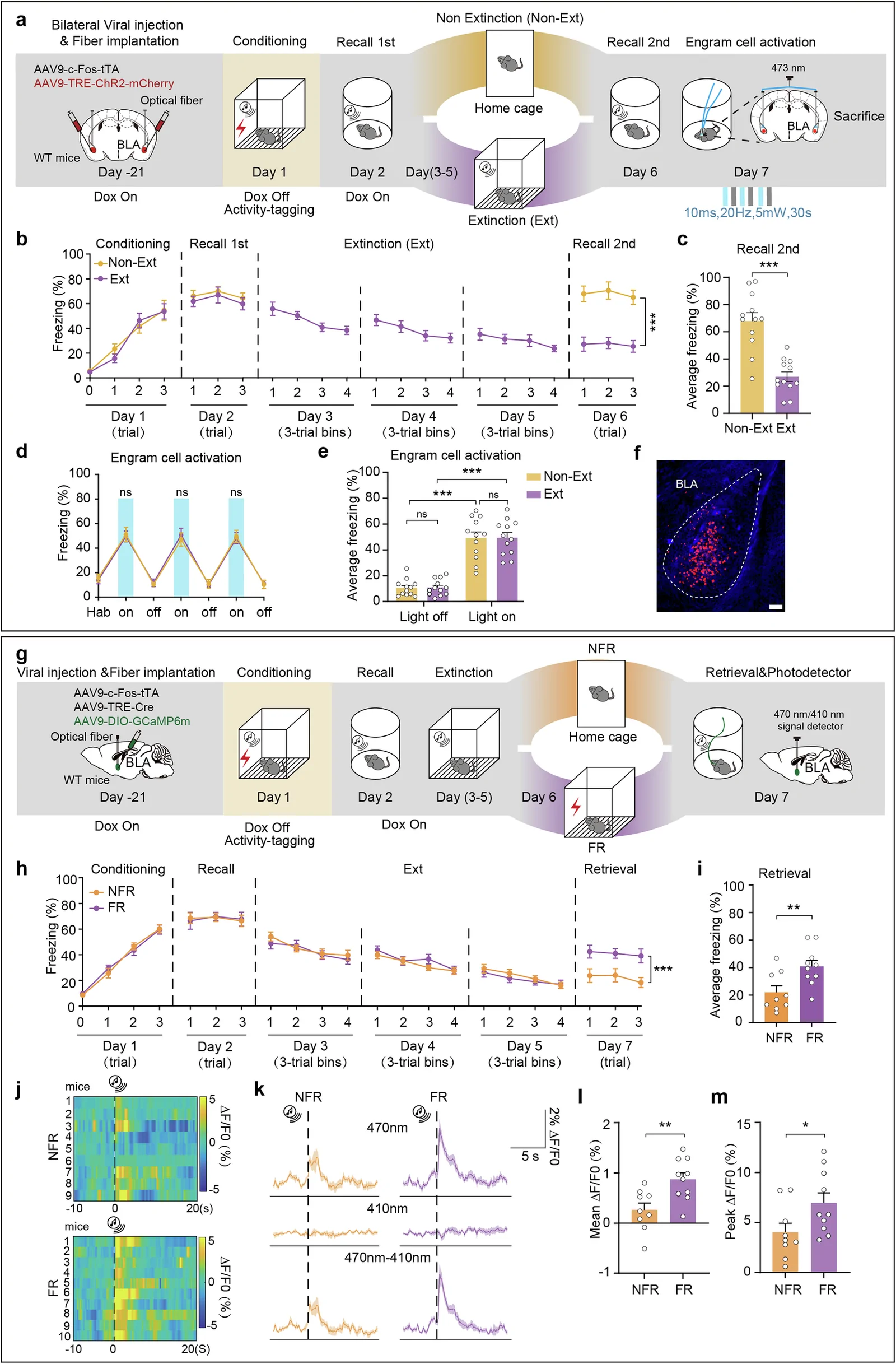
Activation of BLA engram cells evokes relapse of extinguished fear. **a** Experimental design of basolateral amygdala (BLA) engram cells activation by optogenetic manipulation. **b** Freezing levels of conditioning on day 1, first (1st) recall on day 2, extinction during days 3 ~ 5, second (2nd) recall on day 6. Recall 2nd phase, Non-Ext versus Ext: *F*_(1,66)_ = 76.78, *P* < 0.0001; *n* = 12 mice per group. **c** Freezing levels of recall 2nd fear expression after extinction in Non-Ext and Ext groups; (Non-Ext versus Ext, *t* = 5.738, *P* < 0.0001). **d** Freezing levels for the three pairings of light-off and light-on during the blue light stimulation of BLA engram cells (two-way analysis of variance, group factor, Non-Ext versus Ext: *P* = 0.9937; treatment factor, light on versus light off: *P* < 0.0001; interaction: *P* = 0.9921; *n* = 12 mice per group). **e** Average freezing responses for light on and light off epochs in Non-Ext and Ext groups (group factor: *P* = 0.9365, time factor: *P* < 0.0001, interaction: *P* = 0.9823). Light off (Non-Ext versus Ext): *P* > 0.9999; light on (Non-Ext versus Ext): *P* > 0.9999; Non-Ext (light on versus light off): *P* < 0.0001; Ext (light on versus light off): *P* < 0.0001; *n* = 12 mice per group. **f** Representative fluorescence image of mCherry-expressing cells in BLA coronal section (scale bar, 100 µm). **g** Schematic diagram of fiber photometry recordings in BLA engram cells. **h** Freezing levels of cue-induced BLA engram cells reactivity in fear reinstatement (FR) and non-fear reinstatement (NFR) groups (retrieval phase: NFR versus FR: *F*_(1,51)_ = 22.79, *P* < 0.0001; *n* = 12 mice per group). **i** Average levels of freezing response in retrieval phase (NFR versus FR, *t* = 2.939, *P* = 0.0092). **j** Heatmaps showing the calcium fluorescence signals of BLA engram cells in NFR (top; *n* = 9 mice) group and FR group (bottom; *n* = 10 mice) responded to conditioned stimulus (CS) during fear retrieval phase. Black dashed line indicates CS delivery. **k** Peri-event plot of the average calcium fluorescence signals response to CS events. The thick orange (or purple) line represents the average and the orange (or purple)-shaded regions represent the standard error of the mean (NFR: *n* = 9 mice; FR: *n* = 10 mice). **l**,**m** Quantification of mean Δ*F*/*F*_0_ (part **l**) and peak Δ*F*/*F*_0_ (part **m**) during the period of 20 s cue stimulus (NFR: *n* = 9 mice; FR: *n* = 10 mice. **l** *t* = 3.2220, *P* = 0.005; **m** *t* = 2.145, *P* = 0.0467). Statistical analyses were performed by two-way analysis of variance with post hoc Sidak multiple comparisons (parts **b** **d**, **e** and **h**) and two-tailed unpaired *t* test (parts **c**, **i**, **l**, and **m**). Data are shown as mean ± SEM. *P < 0.05, **P < 0.01, ***P < 0.001, and ns for no significance. WT, wild-type.

For dual labeling of c-Fos (Figs. 2 and 3), mice were injected with a mixture of AAV-c-Fos-tTA and AAV-TRE-mCherry to selectively express mCherry protein in the presence of Dox diet (50 mg/kg). The diet returned to Dox-free diet immediately after experiment. All mice were anesthetized with isoflurane 90 min after the fear conditioning procedure, and perfused with saline followed by 4% paraformaldehyde (w/v). Brains were post-fixed in 4% paraformaldehyde for 24 h at 4 °C and then immersed in 30% sucrose (w/v). Coronal sections of 30-μm thickness were cut using a cryostat microtome (CM1860, Leica, Germany) and stored in antifreeze solution at −20 °C in a 24-well plate. Sections were blocked with a solution of 1% BSA, 3% goat serum, and 0.3% Triton X-100 in 0.01 M phosphate-buffered saline (PBS) for 30 min at 37 °C. After washing three times with PBST (PBS with 0.05% Tween 20) for 10 min each, sections were incubated with primary antibodies against TH (1:1,000; mouse anti-TH, T1299, Millipore) or c-Fos (1:1,000; rabbit anti-c-Fos, 2250S, Cell Signaling Technology) in the blocking solution for overnight at 4 °C. Sections were then washed three times with PBST and incubated with secondary antibodies conjugated to Alexa 594 (goat anti-mouse, 1:500; 115-585-003, Jackson ImmunoResearch) or Alexa 488 (goat anti-rabbit, 1:500; 111-545-003, Jackson ImmunoResearch) in the blocking solution for 2 h at room temperature. After rinsing as before, sections were stained with 4′,6-diamidino-2-phenylindole (1:1,000, D9542, Sigma-Aldrich) to label nuclei. Sections were mounted on glass slides and coverslipped with 50% glycerol. For c-Fos staining in LC^TH^ neurons (Fig. 1), the primary antibodies against TH (1:1,000; mouse anti-TH, T1299, Millipore) and c-Fos (1:1,000; rabbit anti-c-Fos, 2250S, Cell Signaling Technology) were employed for double immunofluorescence staining in a similar way. Images were acquired at 10× magnification using an Olympus VS120 microscope (Olympus, Japan). Fiji software was used to quantify c-Fos^+^ and mCherry^+^ cells in each brain slice. Cell counting was performed by two independent researchers who were blind to the experimental conditions.

### Statistical analysis

The unpaired *t* test is used to compare the mean of two independent groups. Repeated-measures one-way analysis of variance (ANOVA) with post hoc Holm–Sidak multiple comparisons is conducted for comparing the same objects measured by more than twice. Also, two-way ANOVA with post hoc Sidak’s or Tukey’s multiple comparisons tests is used to analysis the double factor experiments. The non-parametric test is conducted when the data do not meet the assumptions of the Gaussian distribution analyzed by Kolmogorov–Smirnov test. The statistical analyses were performed using MATLAB or R project, and the figures are generated by Adobe Illustrator. All data are shown as mean ± SEM. ^*^P < 0.05, ^**^P < 0.01, ^***^P < 0.001, and ns for no significance.

## Results

### FR after extinction causes fear relapse and reactivates BLA engram cells

Given that re-exposure to the US (for example, footshock) following fear extinction can partially restore the conditioned response (for example, freezing) in classical fear conditioning^32,33^, we first performed the FR paradigm to simulate fear relapse (Fig. 2a). Following the initial fear conditioning on day 1, the mice demonstrated significant freezing behavior when presented with a CS during recall (Fig. 2b). However, after undergoing a series of extinction sessions from days 3 to 5, this freezing behavior markedly diminished (Fig. 2b). After extinction, the FR mice were exposed to footshock three times in the absence of CS on day 6, whereas the NFR mice were placed to a neutral environment. In the fear retrieval test on day 7, the FR mice showed a higher freezing response compared with the NFR group (Fig. 2b,c), indicating that FR is sufficient to drive the relapse of extinguished fear.

To investigate whether BLA^engram^ is implicated in FR, we used an activity-dependent dual labeling method for memory engram cells in the BLA using the tetracycline-off (Tet-off) system and immunofluorescence assays^34,35^. Briefly, a tetracycline transactivator (tTA) vector (AAV9-c-Fos-tTA) and a tetracycline-responsive promoter element (TRE) vector (AAV9-TRE-mCherry) were injected to label BLA neurons activated by fear conditioning (activity-tagging) in the absence of a doxycycline (Dox) diet (Fig. 2d). This Tet-off strategy for labeling memory engram cells has been validated to ensure that no unintended expression leakage occurred in the presence of a Dox diet (Supplementary Fig. 1). Subsequently, BLA neurons responding to fear retrieval were labeled through immunofluorescence staining using a c-Fos antibody (Fig. 2d). The representative images of BLA^engram^ were presented with mCherry-positive (mCherry^+^) cells shown in red (for the first labeling) and c-Fos-positive (c-Fos^+^) cells shown in green (for the second labeling) (Fig. 2e,f). There was no notable difference in the quantity of mCherry^+^ neurons between FR and NFR groups (Fig. 2g). However, FR mice showed a pronounced increase in c-Fos^+^ cells (Fig. 2h), along with a higher count and percentage of double-positive (mCherry^+^c-Fos^+^) cells (Fig. 2i,j), indicating that FR significantly reactivates BLA memory engram cells.

Taken together, these findings reveal that FR after extinction can lead to a relapse of extinguished fear and that this process is linked to the reactivation of fear memory engram cells in the BLA.

### Post-extinction optogenetic reactivation of BLA engram cells still triggers relapse of extinguished

**Fig. 4:**
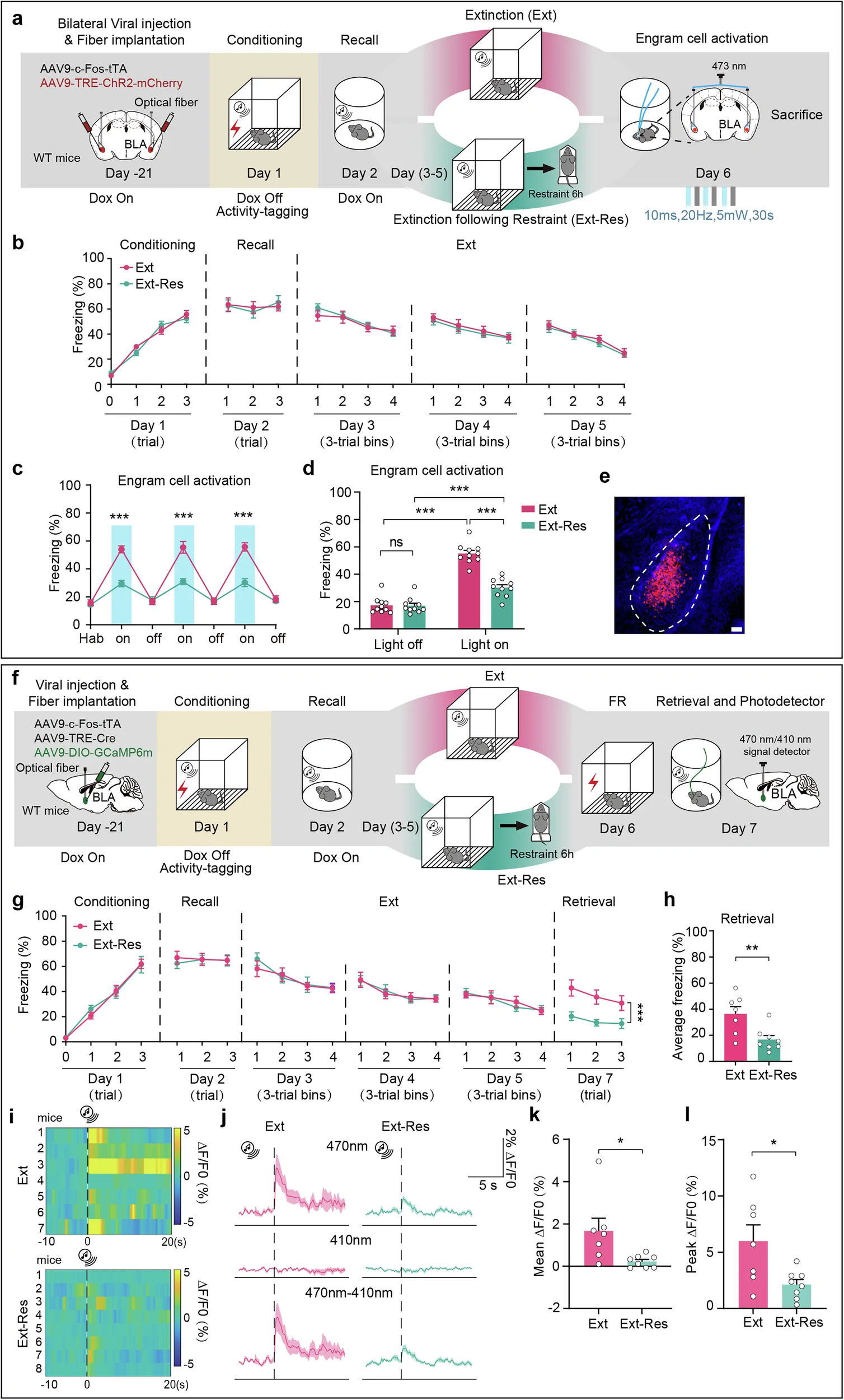
Ext-Res intervention eradicates fear memory encoded in BLA engram cells. **a** Schematic of viral strategy and mouse behavior paradigm. **b** Freezing levels in fear conditioning on day 1, recall on day 2, and extinction during days 3–5. **c** Freezing levels across fear retrieval session after basolateral amygdala (BLA) engram cells stimulation for light on and light off groups (group: *P* < 0.0001, Ext versus Ext-Res; time: *P* < 0.0001; interaction: *P* < 0.0001; *n* = 12 mice per group). **d**, Average freezing levels for light on and light off epochs (group: *P* < 0.0001, time: *P* < 0.0001, interaction: *P* < 0.0001). Light off (Ext versus Ext-Res): *P* = 0.9998; light on (Ext versus Ext-Res): *P* < 0.0001; Ext (light on versus light off): *P* < 0.0001; Ext-Res (light on versus light off): *P* = 0.0068; *n* = 12 mice per group. **e** Representative fluorescence image of BLA mCherry-expressing cells via the doxycycline (Dox)-controlled activity-tagging (scale bar, 100 µm). **f** Experimental design of in vivo fiber photometry recordings in BLA engram cells. **g** Freezing levels in fear conditioning on day 1, recall on day 2, extinction during days 3–5, and retrieval on day 7 (retrieval phase: Ext versus Ext-Res: *F*_(1,39)_ = 26.54, *P* < 0.0001; Ext: *n* = 7 mice; Ext-Res:*n* = 8 mice). **h** Average freezing levels during the retrieval session (Ext versus Ext-Res, *t* = 3.151, *P* = 0.0077). **i** Heatmaps denoting the calcium fluorescence signals of BLA engram cells in Ext mice (top; *n* = 7) and Ext-Res mice (bottom; *n* = 8) responded to conditioned stimulus (CS) in fear retrieval session. Black dashed line represents CS delivery. **j** Peri-event plot of GCaMP6m signals response to CS stimulus (Ext: *n* = 7 mice; Ext-Res: *n* = 8 mice). Mean fluorescence changes (part **k**: *t* = 2.558, *P* = 0.0238) and peak fluorescence changes (part **l**:*t* = 2.694, *P* = 0.0184) after CS delivery in Ext mice (*n* = 7) and Ext-Res mice (*n* = 8). Significant differences were performed by the two-way analysis of variance with post hoc Sidak multiple comparisons (parts **b**–**d**and **g**), and two-tailed unpaired *t* test (parts **h**, **k** and **l**). Data are presented as mean ± SEM. *P < 0.05, **P < 0.01, ***P < 0.001, and ns for no significance. FR, fear reinstatement; TRE, tetracycline-responsive promoter element; tTA, tetracycline transactivator; WT, wild-type.

To directly assess whether extinction paradigm alone could effectively eradicate fear memory encoded in BLA^engram^, all mice were bilaterally injected with AAV9-c-Fos-tTA and AAV9-TRE-ChR2-mCherry. Following a 21-day period of viral expression, BLA^engram^ was labeled during fear conditioning after the withdrawal of the Dox diet. The fear conditioning-related BLA^engram^ was subsequently activated to assess freezing behavior using blue light (473 nm, 10 ms pulse, 20 Hz, 5 mW) across three intermittent sessions (Fig. 4a). Following a 3-day extinction paradigm, a cued recall test was conducted, revealing a significant reduction in freezing behavior among the extinction (Ext) mice (Fig. 4b,c). Despite the successful extinction training, results from the optogenetic manipulations revealed that reactivation of fear conditioning-related BLA^engram^ after extinction led to a marked increase in freezing behavior for both the Ext and non-extinction (Non-Ext) groups (Fig. 4d,e). Confirmation of viral expression was achieved through fluorescence imaging (Fig. 4f and Supplementary Fig. 2). This suggests that extinction training alone does not completely erase fear memories encoded in these engram cells. To exclude the possibility of general fear-related BLA cells, an additional experiment was conducted to assess whether activation of single social defeat stress-labeled BLA cell populations would affect fear conditioning-induced freezing behavior. Results indicated that activation of these single social defeat stress-labeled populations did not result in increased freezing behavior (Supplementary Fig. 3). This evidence supports the notion that the manipulation specifically targeted fear conditioning-related memory engram cells within the BLA, rather than influencing general fear-related cells.

The reactivity of BLA^engram^ to CS was further explored by expressing a genetically encoded fluorescent calcium indicator, GCaMP6m, in these neurons and implementing an FR-based fear relapse paradigm (Fig. 4g). BLA^engram^ in FR mice exhibited higher fluorescence signal changes, both mean Δ*F*/*F*_0_ and peak Δ*F*/*F*_0_, in response to tone stimulation of fear retrieval, compared with those in NFR mice (Fig. 4h–m and Supplementary Fig. 4).

In conclusion, the findings strongly indicate that post-extinction optogenetic activation of fear conditioning-related BLA^engram^ still can trigger the relapse of extinguished fear. This highlights the enduring nature of fear memories, which may remain dormant and are capable of being reactivated even after extinction attempts.

### Ext-Res intervention reduces FR-induced fear relapse and reactivated BLA engram cells

Traumatic memories have the potential to be modified through various methods during a critical period known as the “reconsolidation time window”, which typically lasts up to 6 h post-retrieval. Interventions within this timeframe may provide opportunities for therapeutic strategies aimed at reducing the emotional impact of traumatic memories. We investigated whether the Ext-Res intervention can prevent the FR-induced relapse of extinguished fear. Initially, all mice underwent fear conditioning followed by cued recall tests. The Ext-Res group received 6 h of restraint treatment immediately after extinction from days 3 to 5. Then all the mice were subjected to an FR paradigm and a fear retrieval test (Fig. 3a). The results of fear retrieval tests indicated that Ext-Res mice showed significantly lower freezing level compared with other groups (Fig. 3b,c and Supplementary Fig. 5), indicating that only the combined Ext-Res intervention efficiently ameliorates the relapse of extinguished fear. To determine whether the Ext-Res intervention led to the behavioral consequences, mice underwent a battery of behavioral tests, including open field test, elevated plus maze test, rotarod test, grip strength test, and novel object recognition test. These results suggest that the 6-h restraint had no significant impact on locomotor activity, anxiety-like behavior, or cognitive function (Supplementary Fig. 6 and Supplementary Fig. 7). Furthermore, to determine whether a shorter duration of restraint could produce similar effects, we conducted a 90-min restraint intervention following extinction. Our findings demonstrated that a 90-min restraint was sufficient to alleviate fear relapse (Supplementary Fig. 8).

The activity-dependent dual labeling of c-Fos was utilized to assess the number of reactivated BLA memory engram cells during fear retrieval (Fig. 3d). The number of mCherry^+^ cells showed no significant difference between Ext and Ext-Res groups, indicating that the viral injection results in approximately equal numbers of cells expressing mCherry (Fig. 3e–g). However, Ext-Res mice showed a significant reduction in c-Fos^+^ cells, mCherry^+^c-Fos^+^ cells, and the ratio of mCherry^+^c-Fos^+^ cells among mCherry^+^ cells compared with Ext mice (Fig. 3h–j). These results suggest that the Ext-Res intervention reduces the reactivation of BLA^engram^ during the memory consolidation window, thereby preventing the relapse of extinguished fear.

### Ext-Res intervention efficiently eradicates fear memory encoded in BLA engram cells

**Fig. 5:**
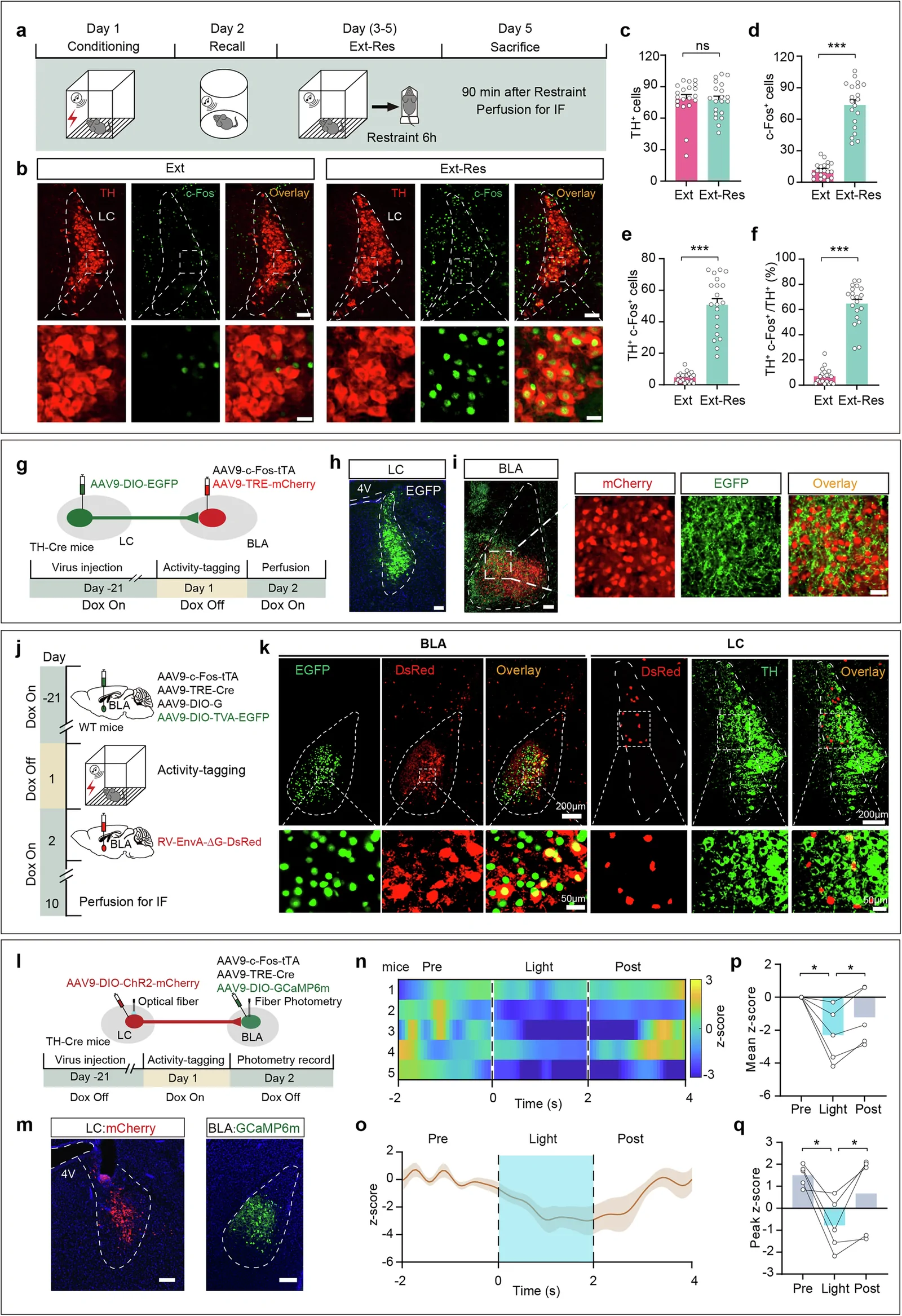
LC^TH^ neurons respond to Ext-Res intervention and project to BLA engram cells. **a** Experimental diagram for c-Fos staining activated by Ext-Res intervention in LC^TH^ neurons. **b** Representative images of tyrosine hydroxylase (TH^+^) (red) and c-Fos^+^ (green) immunofluorescence (IF) in LC^TH^ neurons of Ext mice (left) and Ext-Res mice (right). Top: scale bar, 100 µm; bottom: scale bar, 25 µm. **c**–**f** Quantitative analysis of TH^+^ cells (part **c**: *t* = 0.1936, *P* = 0.8476), c-Fos^+^ cells (part **d**: *t* = 12.15, *P* < 0.0001), and dual labeling TH^+^c-Fos^+^ overlap cells (part **e**: *t* = 11.33, *P* < 0.0001) in Ext and Ext-Res mice. Percentage of dual labeling TH^+^c-Fos^+^ cells in TH^+^ cells in each group (part **f**: *t* = 15.33, *P* < 0.0001). For **c**–**f** there are 20 slices from 5 mice in each group. **g** Virus strategy for anterograde tracing the circuit from LC^TH^ neurons to basolateral amygdala (BLA) engram cells. **h** Fluorescence images of EGFP^+^ cells in locus coeruleus (LC). Scale bar, 100 µm. **i**, Fluorescence images of EGFP^+^ projections and mCherry^+^ cells in BLA. Scale bar, 100 µm (left). Topical enlarge: scale bar, 50 µm (right). **j** Experimental paradigm for trans-monosynaptic retrograde tracing. **k** Fluorescence images of BLA and LC. Scale bar, 200 µm for upper panels and scale bar, 50 µm for lower panels. **l** Schematic of viral strategy and optical fibers implantation. **m** Representative images of LC (red) and BLA (green). **n** Heatmap of BLA GCaMP6m-expressing cells responded to optical stimulation with blue light (473 nm, 20 Hz, 10 ms pulse width, 2 s duration, 20 s interval). **o** Traces of fluorescence dynamics following blue light stimulation of ChR2-expressing cells in LC (*n* = 5 mice). Mean *z*-score (part **p**) and peak *z*-score (part **q**) of fluorescence signals in pre-light session (pre group), light stimulation (light), and post-light session (post group). For mean *z*-score, light versus pre: *P* = 0.0357; light versus post: *P* = 0.0128; *n* = 5 mice. For peak *z*-score, light versus pre: *P* = 0.0358, light versus post: *P* = 0.0427; *n* = 5 mice. Statistical analyses were conducted by two-tailed unpaired *t* test (parts **c**–**f**, **p** and **q**). Data are shown as mean ± SEM. *P < 0.05, **P < 0.01, ***P < 0.001, and ns for no significance. EGFP enhanced green fluorescent protein, Ext-Res, extinction-restraint, FR, fear reinstatement, TRE, tetracycline-responsive promoter element, tTA, tetracycline transactivator, WT, wild-type.

To assess the impact of the Ext-Res intervention on fear memory encoded in BLA^engram^, we used the c-Fos-tTA and TRE strategy to label and manipulate fear conditioning-activated BLA neuronal ensembles. All the mice were bilaterally injected with a viral cocktail (AAV9-c-Fos-tTA and AAV9-TRE-ChR2-mCherry). After 3 weeks of viral expression, the activity-dependent BLA neuronal ensembles in response to fear conditioning were labeled in the absence of a Dox diet. Both the Ext-Res mice and Ext mice received blue light stimulation (Fig. 5a). The photoactivation-induced freezing response during retrieval was used as a quantifiable measure of the fear memory encoded in BLA^engram^.

The Ext-Res group exhibited behavioral patterns similar to the Ext group during conditioning, recall, and extinction phases (Fig. 5b). However, during the light-on sessions, the Ext-Res group demonstrated a significantly lower freezing response than the Ext group (Fig. 5c,d). This indicates that the optogenetic activation of BLA^engram^ cannot fully trigger the relapse of extinguished fear following the Ext-Res intervention. The injection site and viral expression were confirmed by fluorescence imaging of the BLA area (Fig. 5e and Supplementary Fig. 9). These experiments support the notion that Ext-Res intervention effectively reduces fear relapse by erasing the fear memory encoded in BLA^engram^.

Furthermore, we examined whether Ext-Res intervention reduces the reactivation of BLA^engram^ using GCaMP6m labeling via an intersectional viral approach. Optical fibers were bilaterally implanted above the BLA injection sites. After the Ext-Res intervention and FR-based fear relapse paradigm, mice were placed in a neutral environment to image the calcium responses of BLA^engram^ to CS using fiber photometry (Fig. 5f). The behavioral performance of Ext-Res or Ext mice was consistent with the results from previous experiments (Fig. 5g,h). BLA^engram^ exhibited time-locked responses to conditioned sound stimulation, with significantly lower average and peak responses in Ext-Res mice compared with Ext mice (Fig. 5i–l; and Supplementary Fig. 10). These results suggest that Ext-Res intervention can erase fear memory during the reconsolidation time window and reduce the reactivation of BLA^engram^ in response to CS during fear retrieval.

### LC^TH^-BLA^engram^ circuit participates in preventing fear relapse following Ext-Res intervention

The LC neurons are known to be closely associated with stress responses, projecting primarily to various subnuclei of the amygdala, particularly the BLA^13,14,16,17^. In this study, we first investigated whether TH positive-LC neurons (LC^TH^) are activated by Ext-Res intervention by employing double immunofluorescence staining (Fig. 1a). Brain slices containing the LC area were double-stained with anti-TH for red and anti-c-Fos for green after Ext or Ext-Res intervention (Fig. 1b). The results showed that the Ext-Res mice had a significantly higher number of c-Fos^+^ cells, TH^+^c-Fos^+^ cells, and an increased percentage of TH^+^c-Fos^+^ cells in TH^+^ cells compared with the Ext group mice, indicating that LC^TH^ neurons are specifically activated by the Ext-Res intervention, but not by Ext only (Fig. 1c–f).

Next, we explored the structural connectivity of the circuit from LC^TH^ neurons to fear conditioning-activated BLA^engram^ by viral tracing. For anterograde tracing, TH-Cre transgenic mice were injected with AAV9-DIO-EGFP into the LC, whereas a mixture of AAV9-c-Fos-tTA and AAV9-TRE-mCherry was administered into BLA (Fig. 1g). The representative image of LC showed that the EGFP-positive (EGFP^+^) cells were strictly resident within LC (Fig. 1h). Meanwhile, enlarged fluorescence images from the BLA indicated that mCherry^+^ cells were surrounded by EGFP^+^ projections, suggesting a direct projection from LC^TH^ to BLA^engram^ (Fig. 1i). For trans-monosynaptic retrograde tracing, a mixture of AAV9-c-Fos-tTA, AAV9-TRE-Cre, and helper virus (AAV9-DIO-TVA-EGFP and AAV9-DIO-G) was administered into BLA. After 3 weeks, the BLA^engram^ was labeled by fear conditioning during Dox-off period, following by RV-EnvA-ΔG-DsRed injection in BLA (Fig. 1j). Immunostaining results from this trans-monosynaptic retrograde tracing confirmed that BLA^engram^ received the direct projections from LC^TH^ neurons (Fig. 1k).

Additionally, we assessed the functional connectivity of an LC^TH^-BLA^engram^ circuit by a novel approach that combined simultaneous fiber photometry recording and optogenetic stimulation. The calcium indicator GCaMP6m was selectively expressed in fear conditioning-activated BLA^engram^ cells using an intersectional viral strategy, whereas AAV9-DIO-ChR2-mCherry was used to express opsins specifically in LC^TH^ neurons in TH-Cre transgenic mice. Optical fibers for optogenetic stimulation and fiber photometry were placed above the LC and BLA injection sites, respectively (Fig. 1l). Following a 3-week recovery, mice underwent fear conditioning to label BLA^engram^ with Dox-off diet. This strategy ensured that ChR2-mCherry was selectively expressed in LC^TH^ neurons, and GCaMP6m expressed in BLA^engram^ (Fig. 1m). Compared with the pre-light and post-light session, optogenetic activation of ChR2-expressing LC^TH^ neurons with blue light stimulation generally resulted in decreased calcium fluorescence signal changes, indicating that LC^TH^ activation leads to suppression of BLA^engram^ (Fig. 1n–q).

In summary, these findings suggest that the LC^TH^-BLA^engram^ circuit has a critical role in preventing the relapse of extinguished fear by Ext-Res intervention.

### Bidirectional modulation of the LC-BLA circuit mimics or reverses the therapeutic effect of Ext-Res intervention against the FR-induced fear relapse

**Fig. 6:**
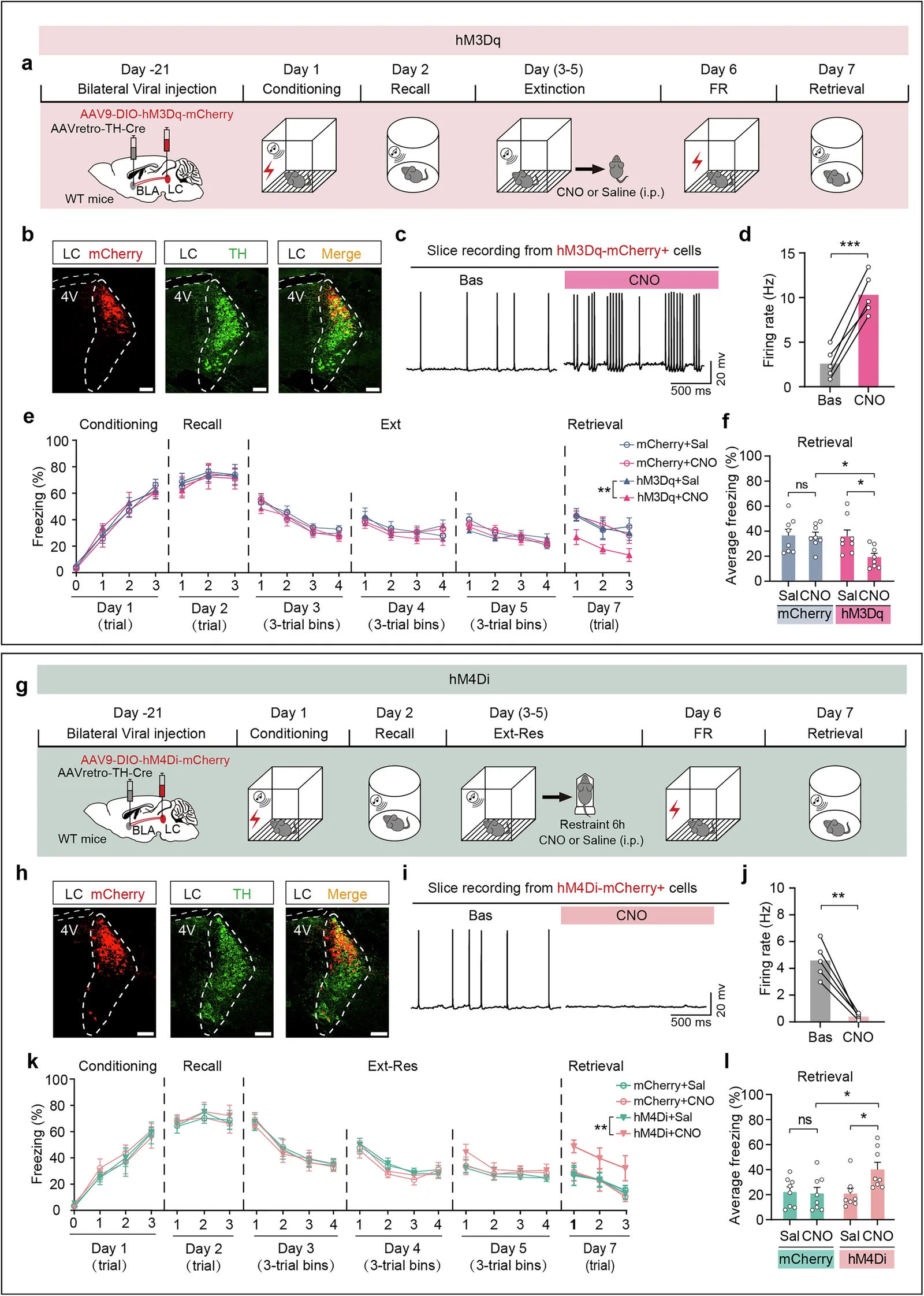
Activation or inhibition of LC^TH^-BLA circuit mimics or reverses the effect of Ext-Res intervention. **a** Experimental paradigm for chemogenetics activation of LC^TH^-BLA circuit. **b** Example of locus coeruleus (LC) image showing colocalization of hM3Dq-mCherry with tyrosine hydroxylase (TH^+^). Scale bar, 100 µm. Example traces showing CNO-increased action potential firing in hM3Dq-expressing TH^+^ neurons in LC (part **c**) and quantitative analyses of firing rate (part **d**). **e** Freezing levels of fear response across the conditioning (day 1), recall (day 2), extinction (days 3~5), and retrieval (day 7). Retrieval phase: group factor: *P* = 0.0004; time factor: *P* = 0.0063; mCherry (NS versus CNO): *P* = 0.9992, hM3Dq (NS versus CNO): *P* = 0.0028, NS (mCherry versus hM3Dq): *P* = 0.9983, CNO (mCherry versus hM3Dq): *P* = 0.00247; *n* = 8 mice per groups. **f** Average freezing levels during the fear retrieval phase (group factor: *P* = 0.0483, treatment factor: *P* = 0.0527, interaction: *P* = 0.0701; mCherry (NS versus CNO): *P* = 0.9996; hM3Dq (NS versus CNO): *P* = 0.0466; NS (mCherry versus hM3Dq): *P* = 0.9992; CNO (mCherry versus hM3Dq): *P* = 0.0437; *n* = 8 mice per group). **g** Virus strategy for chemogenetic manipulation. **h** Example of LC image showing colocalization of hM4Di-mCherry with TH^+^. Scale bar = 100 µm. Example traces showing CNO-decreased action potential firing in hM4Di-expressing TH^+^ neurons in LC (part **i**) and quantitative analyses of firing rate (part **j**). **k** Freezing behavior in four groups across the conditioning (day 1), recall (day 2), extinction (days 3~5), and retrieval (day 7). Retrieval phase: group: *P* = 0.0005; time: *P* = 0.0052; mCherry (NS versus CNO): *P* = 0.9942, hM4Di (NS versus CNO): *P* = 0.0025, NS (mCherry versus hM4Di): *P* = 0.9965, CNO (mCherry versus hM4Di): *P* = 0.0022; *n* = 8 mice for each group. **l** Mean freezing levels during the fear retrieval session levels after chemogenetic inhibition of LC^TH^-BLA circuit. Group factor: *P* = 0.0696; treatment factor: *P* = 0.0677; interaction: *P* = 0.0415; mCherry (NS versus CNO): *P* = 0.9983, hM4Di (NS versus CNO): *P* = 0.0379, NS (mCherry versus hM4Di): *P* = 0.998, CNO (mCherry versus hM4Di): *P* = 0.0387; *n* = 8 mice per group. Significant differences were assessed by the two-way analysis of variance with post hoc Tukey’s multiple comparisons (parts **e**, **f**, **k** and **l**), and two-tailed paired *t* test (parts **d**and **j**). Data are displayed as mean ± SEM. *P < 0.05, **P < 0.01, ***P < 0.001, and ns for no significance. CNO, clozapine-*N*-oxide; Ext-Res, extinction-restraint; FR, fear reinstatement; NS, normal saline; WT, wild-type.

To test our hypothesis regarding the role of LC-BLA circuit in fear relapse, we aimed to artificially manipulate this circuit using chemogenetic tools during the critical memory reconsolidation window. First, we investigated whether activating the LC-BLA circuit mimics the therapeutic effects of Ext-Res intervention on preventing the relapse of extinguished fear. To selectively express the excitatory DREADD (designer receptor exclusively activated by designer drug) hM3Dq in BLA-projecting LC^TH^ neurons, mice were injected bilaterally with AAVretro-TH-Cre into BLA and AAV9-DIO-hM3Dq-mCherry (hM3Dq mice) or AAV9-DIO-mCherry (mCherry mice) into LC (Fig. 6a). The hM3Dq-mCherry expression and the efficiency of chemogenetic activation were validated by immunofluorescence staining and electrophysiological recordings, respectively (Fig. 6b–d). Immediately following extinction training, LC^TH^-BLA circuit was chemogenetically activated by intraperitoneal injection of the DREADD agonist CNO. Mice in the hM3Dq+CNO group exhibited a significantly lower freezing response during fear retrieval than those in the other three groups (Fig. 6e,f). This finding indicates that activating the LC^TH^-BLA circuit is sufficient to replicate the therapeutic effects of Ext-Res intervention against fear relapse.

Next, we examined whether inhibiting the LC^TH^-BLA circuit could reverse the benefits of Ext-Res intervention in preventing fear relapse. The inhibitory DREADD hM4Di was expressed specifically in BLA-projecting LC^TH^ neurons by injection of AAVretro-TH-Cre in BLA and AAV9-DIO-hM4Di-mCherry (hM4Di group) or AAV9-DIO-mCherry (mCherry group) in LC (Fig. 6g). Similar to the excitatory DREADD, we validated hM4Di-mCherry expression and suppression efficacy through immunofluorescence and electrophysiological methods (Fig. 6h–j). Mice in the hM4Di+CNO group showed significantly higher freezing levels compared with other groups (Fig. 6k,l), suggesting that inhibiting the LC^TH^-BLA circuit can reverse the therapeutic effects of Ext-Res intervention. Together, these results underscore that the LC-BLA circuit is both necessary and sufficient for the therapeutic effects of Ext-Res intervention on relapse of extinguished fear.

### Dopamine, but not norepinephrine, is involved in the therapeutic effects of the Ext-Res intervention on fear relapse

**Fig. 7:**
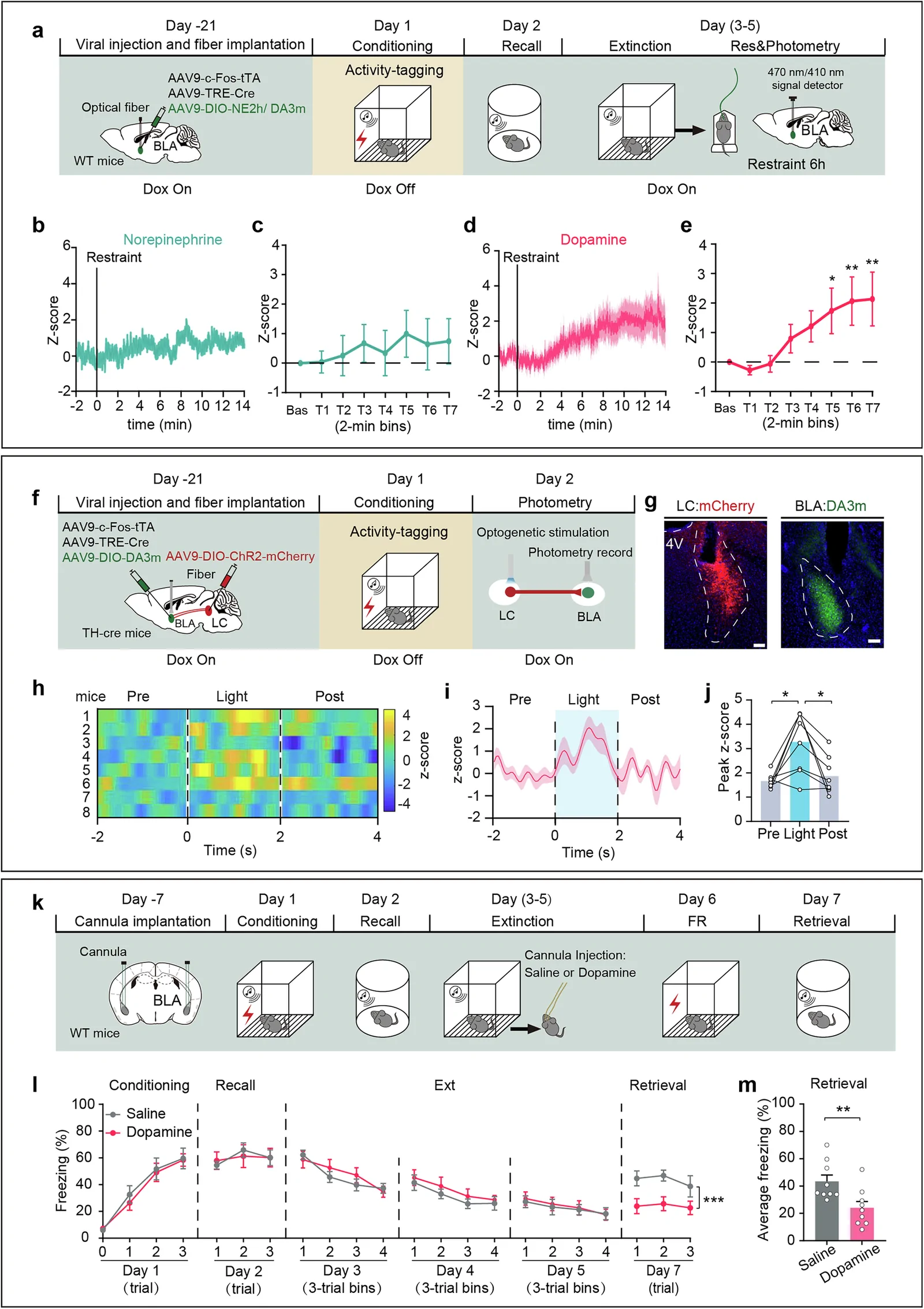
Elevated levels of dopamine in BLA, induced by Res, effectively prevent fear relapse. **a** Experimental paradigm for fiber photometry recordings of genetically encoded G protein-coupled receptor activation-based sensors NE2h and DA3m in basolateral amygdala (BLA) engram cell during restraint treatment. **b** Traces of norepinephrine fluorescence dynamics during the session before restraint and after restraint treatment. **c** Mean response for norepinephrine fluorescence signals *z*-score for 2 min-bins during restraint. **d** Traces of dopamine fluorescence dynamics during the session before restraint and after restraint treatment. **e** Mean response for dopamine fluorescence signals *z*-score for 2 min-bins during restraint (T5–T7 versus bas: *P* = 0.0353; *P* = 0.0076; *P* = 0.0053, *n* = 14 mice.) **f** Experimental diagram for LC TH^+^ neurons activation while recording dopamine release in BLA engram cells. **g** Representative images of LC mCherry^+^ cells (red) and BLA DA3m^+^ cells (green). Scale bar, 100 µm. **h** Heatmaps of the averaged DA3m fluorescence signals (*z*-score) in response to photo stimulation of LC (473 nm laser, a train of 2 s in 20-ms light pulses at 20 Hz). *n* = 8 mice. **i**,**j** Quantitative analysis of DA3m fluorescence signals before and after the light stimulation. **k** Experimental procedures for cannula implantation and dopamine infusion following fear extinction. **l** Behavioral performance of freezing levels across the conditioning on day 1, recall on day 2, extinction during days 3–5, and retrieval on day 7 (retrieval phase: dopamine versus saline, *F*_(1,48)_ = 17.02, *P* = 0.0001; *n* = 9 mice per group). **m** Mean freezing levels during fear retrieval phase (dopamine versus saline: *t* = 2.943, *P* = 0.0095). Statistical analyses were conducted by one-way analysis of variance (ANOVA) (parts **c**, **e** and **j**), two-way ANOVA with post hoc Sidak multiple comparisons (part **l**), and two-tailed unpaired *t* test (part **m**). Data are showed as mean ± SEM. *P < 0.05, **P < 0.01, ***P < 0.001, and ns for no significance. CL, locus coeruleus; FR, fear reinstatement; TH, tyrosine hydroxylase; TRE, tetracycline-responsive promoter element; tTA, tetracycline transactivator; WT, wild-type.

Next, we examined which neurotransmitters are involved in the therapeutic effects of the Ext-Res intervention on fear relapse. We injected a viral cocktail containing AAV9-c-Fos-tTA, AAV9-TRE-Cre, and either AAV9-DIO-DA3m or AAV9-DIO-NE2h into the BLA to selectively express dopamine or norepinephrine receptor probe in BLA^engram^. After activity tagging with fear conditioning on Dox-off diet, we measured calcium fluorescence signal changes in BLA^engram^ via fiber photometry during the restraint treatment period (Fig. 7a). The results indicated no significant changes in norepinephrine levels in post-restraint session compared with those in pre-restraint session. By contrast, dopamine fluorescence signal changes increased significantly (Fig. 7b–e), demonstrating that restraint effectively induces dopamine release to BLA^engram^.

Additionally, we explored the impact of LC^TH^-BLA circuit activation on dopamine release to the BLA^engram^. Dopamine receptor probe DA3m was selectively expressed in BLA^engram^ using the Tet-off strategy, whereas AAV9-DIO-ChR2-mCherry was used to express opsins specifically in LC^TH^ neurons in TH-Cre transgenic mice. Optical fibers for optogenetic activation and fiber photometry were implanted above the LC and BLA injection sites, respectively (Fig. 7f,g). Following a 3-week recovery, mice underwent fear conditioning to label BLA^engram^ with Dox-off diet. This strategy ensured that ChR2-mCherry was selectively expressed in LC^TH^, and DA3m expressed in BLA^engram^. We observed that optogenetic activation of LC^TH^ neurons significantly increased dopamine release to BLA^engram^ cells (Fig. 7h–j).

Furthermore, we assessed the effect of increasing dopamine levels in the BLA via a cannula through intracranial microinjections immediately after fear extinction training (Fig. 7k). Mice receiving post-extinction dopamine injection exhibited a markedly reduced freezing response compared with the saline group (Fig. 7l,m). These findings suggest that administering dopamine directly into the BLA after extinction effectively protects against the relapse of extinguished fear.

### Ext-Res attenuates fear relapse via the dopamine D2 receptors at the LC^TH^-BLA circuit

**Fig. 8:**
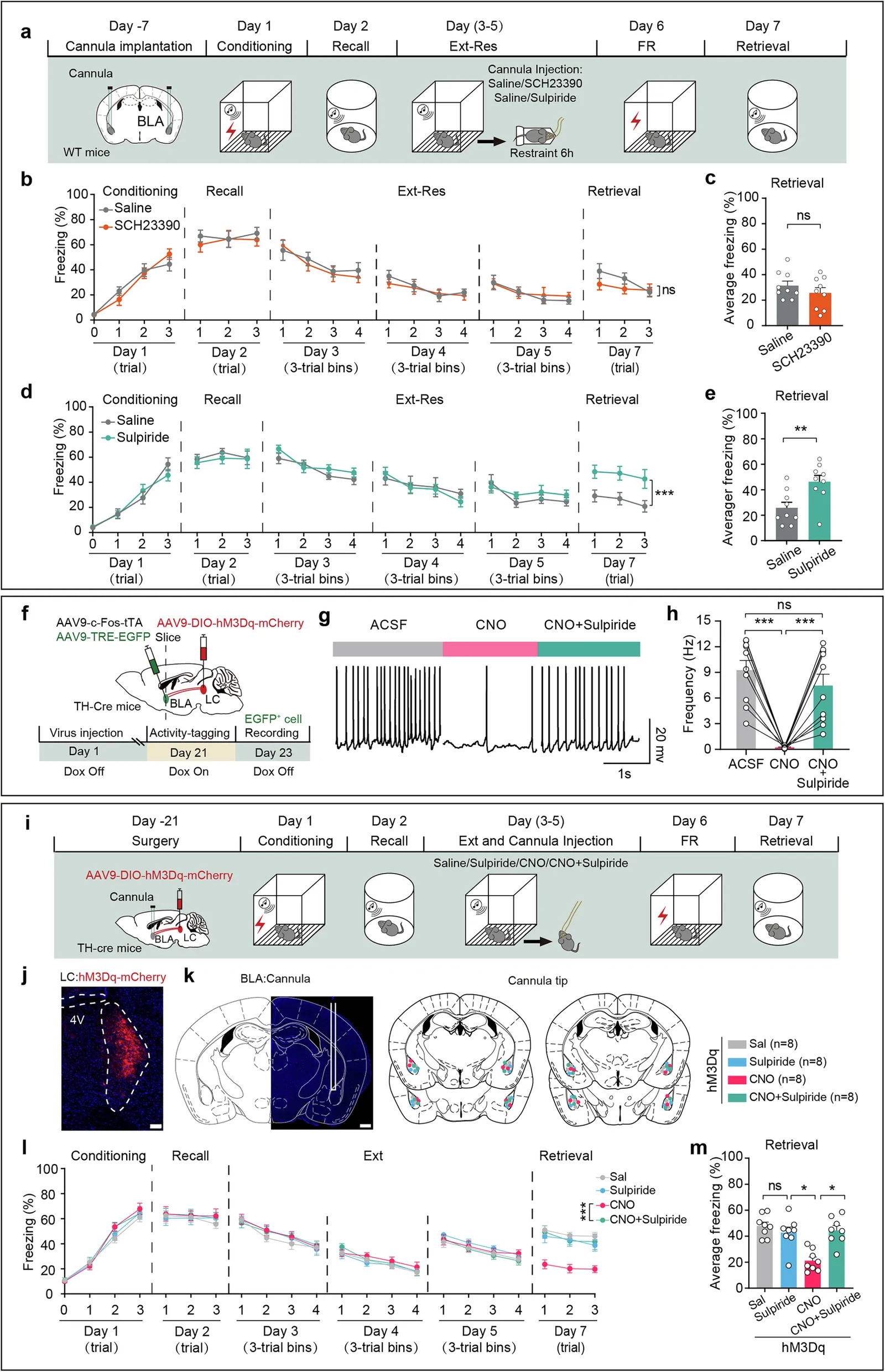
Ext-Res attenuates fear relapse via the dopamine D2 receptor at the LC^TH^-BLA circuit. **a** Schematic representation of the behavioral schedule and dopamine receptors antagonist administration. **b** Freezing levels after basolateral amygdala (BLA) infusion of dopamine D1 receptors antagonist SCH23390 across the conditioning (day 1), recall (day 2), extinction (days 3~5), and retrieval (day 7). Retrieval phase (SCH23390 versus saline): *F*_(1,48)_ = 2.287, *P* = 0.1370; *n* = 9 mice per group. **c** Freezing levels during the fear retrieval phase (SCH23390 versus saline: *t* = 1.042, *P* = 0.3129). **d** Levels of freezing responses after BLA infusion of dopamine D2 receptors antagonist sulpiride (retrieval phase: sulpiride versus saline, *F*_(1,48)_ = 20.58, *P* < 0.0001; *n* = 9 mice per group). **e** Average freezing levels during the fear retrieval session (sulpiride versus saline: *t* = 3.068, *P* = 0.0074). **f** Experimental program for electrophysiology recordings in vitro. Representative traces with ACSF, CNO, and CNO+sulpiride treatment in BLA engram cells (part **g**). Statistical results of BLA engram cell firing rates after chemogenetic activation of LC TH^+^ terminals in different conditions (part **h**). **i** Schematic of viral injection strategy. AAV-DIO-hM3Dq-mCherry was injected into the LC and the dopamine D2 receptor antagonist sulpiride was injected in the BLA after extinction. The viral expression in the LC. Scale bar, 100 µm (part **j**). Infusion sites of BLA (scale bar = 500 µm) and the placements of bilateral cannula tips (part **k**). **l**, Statistical analysis of freezing levels in four groups (*n* = 8 mice for each group). Retrieval phase: group factor: *P* < 0.0001; interaction: *P* = 0.9949; hM3Dq (Sal versus sulpiride): *P* = 0.4600; hM3Dq (Sal versus CNO): *P* < 0.0001; hM3Dq (CNO versus CNO + sulpiride): *P* < 0.0001. **m** Average freezing levels during the fear retrieval phase (*n* = 8 mice for each group). hM3Dq (Sal versus sulpiride): *P* = 0.4611; hM3Dq (Sal versus CNO): *P* < 0.0001; hM3Dq (CNO versus CNO + Sulpiride): *P* = 0.0123. Statistical analyses were conducted by two-way analysis of variance (ANOVA) with post hoc Sidak multiple comparisons, two-tailed unpaired *t* test, and one-way ANOVA. Data are shown as mean ± SEM. *P < 0.05, **P < 0.01, ***P < 0.001, and ns for no significance. ACSF, artificial cerebrospinal fluid; EGFP, enhanced green fluorescent protein; Ext-Res, extinction-restraint; FR, fear reinstatement; TH, tyrosine hydroxylase; TRE, tetracycline-responsive promoter element; tTA, tetracycline transactivator; WT, wild-type.

To better understand the postsynaptic mechanism of dopamine release during the Ext-Res intervention, we administered saline, SCH23390 (a potent antagonist of dopamine D1 receptor) or sulpiride (a selective antagonist of dopamine D2 receptor) into the BLA though a cannula immediately following fear extinction training (Fig. 8a). The D1 receptors antagonist SCH23390 did not significantly affect the impact of the Ext-Res intervention on fear relapse (Fig. 8b,c). However, mice receiving D2 receptors antagonist sulpiride showed a significantly increased freezing level (Fig. 8d,e), suggesting that the Ext-Res intervention alleviates fear relapse in a dopamine D2 receptor-dependent manner.

To further assess the role of D2 receptors in modulating fear memory through the LC-BLA circuit, we expressed hM3Dq in LC^TH^ neurons while labeling BLA^engram^ using the Dox-off system (Fig. 8f). In vitro electrophysiology recordings showed that D2R antagonist sulpiride effectively blocked the CNO-induced decrease in firing rates of BLA^engram^, whereas non-tagged cells exhibited no such change (Fig. 8g,h and Supplementary Fig. 11). Next, we expanded our behavioral experiments by administering the D2R antagonist sulpiride via cannula into the BLA during chemogenetic activation of the LC^TH^ neurons (Fig. 8i). Our results showed that chemogenetic activation of the LC^TH^-BLA circuit led to a significant reduction in freezing behavior during retrieval, whereas D2R inhibition significantly reversed the effects of LC^TH^-BLA circuit activation (Fig. 8j–m). Finally, we evaluated the effects of D2R antagonist sulpiride on anxiety-like behaviors. The data demonstrate that the D2R inhibition does not significantly influence anxiety-like behaviors in the open field and elevated plus maze tests, supporting the specificity of our observations to fear-related processes rather than generalized anxiety (Supplementary Fig. 12). Overall, these results highlight the critical role of D2 receptors in BLA^engram^ in mediating the Ext-Res intervention’s effects on fear memory, supporting the notion that dopamine serves as a key regulator in these processes.

## Discussion

**Fig. 9:**
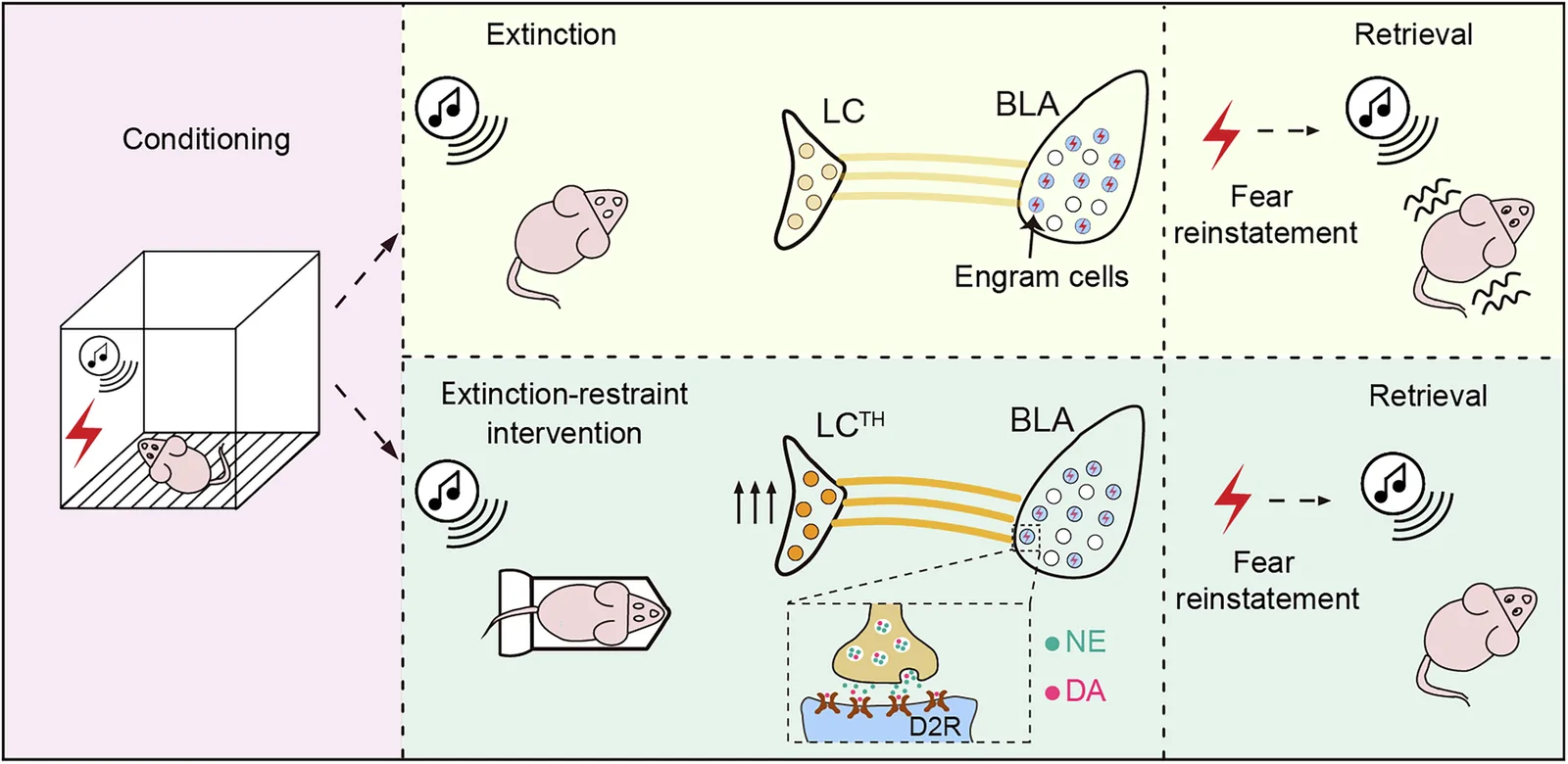
Schematic of restraint intervention-induced LC^TH^ activation suppressing basolateral amygdala (BLA) engrams via D2 signaling to prevent fear relapse. DA, dopamine; LC, locus coeruleus; NE, norepinephrine; TH, tyrosine hydroxylase.

Although exposure therapy has been shown to be effective in treating fear or PTSD^36–38^, preventing the recurrence of fear after treatment remains a great challenge. Here, we show that acute restraint stress following extinction was effective to erase fear memory during the memory reconsolidation time window (Fig. 9). Meanwhile, this therapeutic effect was mediated by the dopaminergic projections from the LC^TH^ neurons to the BLA^engram^. We found that Ext-Res intervention significantly activated LC^TH^ neurons, which send directly projections into BLA^engram^. Activation of LC^TH^-BLA circuit or infusion of DA in BLA mimicked the reduced relapse of extinguished fear by Ext-Res intervention, whereas blockade of the BLA dopamine D2 receptor remarkably abolished this therapeutic effect. Our study has revealed an unexpected role of BLA^engram^ in fear memory erasure by Ext-Res intervention and provided new combination of therapies (such as restraint following exposure therapy) for PTSD relapse prevention. Although the clinical applicability of restraint stress remains limited, this study identifies a dopamine D2 receptor-dependent LC-BLA circuit as a novel therapeutic target.

Fear extinction training in animals provides an excellent experimental analogue for exposure therapy^39^. Similar to exposure therapy, fear extinction involves the repeated presentation of a CS stimulus in the absence of an US, resulting in reduced fear response^40^. However, FR causes fear relapse in spite of successful freezing levels reduction through extinction-based exposure therapy^27^. Consistent with this, we also found extinction alone cannot decrease the FR-induced relapse of extinguished fear and reduce the reactivity of BLA engrams cells labeled by activity-tagging (Fig. 2). Optogenetic activation BLA^engram^ can directly evoke freezing response after extinction (Fig. 4). This is consistent with previous study, which has found that fear extinction alone indeed cannot disrupt the memory reconsolidation to erase fear memory trace^41–43^. Therefore, we provided new evidence that BLA^engram^ encoded and stored fear memory. Chronic stress has been shown to impair cognition, whereas acute stress contributes to improve learning and memory performance^44^. For example, acute stress can lead to amygdala-dependent associative learning enhancement^45^. Therefore, we focussed on whether acute stress following fear extinction training during the memory reconsolidation time window reduces relapse of extinguished fear. Our results indicate that restraint treatment within the 6 h reconsolidation window after extinction training significantly erases the fear memory trace and reduces the reactivity of BLA^engram^, thereby effectively preventing relapse of extinguished fear (Figs. 3 and 5). The effects of stress on memory processes are complex, which depend on the stimulation type, intensity, and duration of the stress. Further study needs to be performed to identify the duration threshold of restraint intervention for the relapse of extinguished fear.

It is well known that LC^TH^ neurons send direct projection to BLA and these projections involve in anxiety-like behavior and fear extinction learning^13,14^. In contrast to these findings, however, no evidence of direct inputs to BLA^engram^ was produced. In this study, the structural connectivity and functional connectivity of the circuit from LC^TH^ neurons to BLA^engram^ were showed by viral tracing and fiber photometry (Fig. 1). We also found that LC^TH^ neurons can be significantly activated by Ext-Res intervention. This finding is consistent with previously published studies, which showed that acute restraint stress obviously activates LC^TH^ neurons^15,20,46^. Furthermore, we provided an undescribed circuit mechanism of LC^TH^-BLA circuit that is both necessary and sufficient for the therapeutic effect of Ext-Res intervention on relapse of extinguished fear. Interestingly, we found that activation of LC^TH^ neurons significantly inhibits the activity of BLA^engram^ (Fig. 1l–q). Dopamine has effects that are both excitatory and inhibitory, which depends on its postsynaptic receptors such as D1 receptor and D2 receptor^22,27^. In this study, pharmacological blockade of dopamine D2 receptors remarkably reverses the therapeutic effect of Ext-Res intervention on relapse of extinguished fear, whereas the blockade of dopamine D1 receptors has no significant effect (Fig. 8). These data must be interpreted with caution because excessive dopamine injections may lead to significant behavioral changes. Overall, these results consistently indicate that Ext-Res intervention erases fear memory trace via dopamine and its D2 receptors signaling pathway.

FR has been proposed to arise from multiple exclusive mechanisms, including the reactivation of the original fear engram^6,7^ as well as the disruption of extinction-related inhibitory control over amygdala output^47^. Here, we demonstrate that the Ext-Res intervention eradicates fear memory encoded in BLA engram cells through D2 receptor-mediated dopaminergic modulation from LC. Mechanistically, the Ext-Res intervention initially reactivates the original fear memory, rendering it labile during the memory reconsolidation window. Subsequent restraint activates LC^TH^ neurons, triggering dopamine release onto BLA engram cells via D2 receptor activation. We propose that, compared with extinction alone, this D2 receptor signaling during reconsolidation induces greater synaptic weakening and additional cellular plasticity changes, ultimately eradicating the original fear memory trace. In addition to the LC inputs to BLA, recent studies have shown that prefrontal cortical inputs to the BLA have a crucial role in fear extinction^48–51^. Future work should therefore investigate whether these prefrontal–BLA circuits also contribute to the therapeutic effect of Ext-Res intervention on fear memory.

Previous studies have demonstrated that stress induces LC activation and NE elevation across the BLA, thereby impairing extinction learning^13,14,52^. These effects were typically measured using bulk approaches (for example, microdialysis), which reflect global neuro-modulatory states during the stress phase. By contrast, our study focusses on the BLA^engram^ subpopulation specifically during the Ext-Res intervention, which occurs within the memory reconsolidation time window. LC projections are not strictly noradrenergic, accumulating evidence shows that LC terminals can generate detectable DA signals and exert DA receptor-mediated effects in several limbic and thalamic regions^19,22^. Owing to the comparatively low expression levels of the dopamine transporter in the BLA region relative to the norepinephrine transporter, this area may also be more susceptible to the formation of DA-enriched microdomains^53,54^. Our results reveal a spatiotemporal dissociation between NE and DA signaling in the BLA during the Ext-Res window. Although restraint stress during Ext-Res elicits a rapid and spatially widespread NE transient at the bulk BLA level, the DA signaling accumulates more slowly and is selectively enriched within the BLA^engram^ population, where it exerts a sustained influence on memory expression (Fig. 7a–e and Supplementary Fig. 13). These findings suggest that LC activation engages dual neuromodulatory modes in the BLA: a fast, global NE signal that may reflect general arousal or stress responses, and a slower, spatially restricted DA signal that preferentially modulates behaviorally relevant memory traces. Moreover, it is well established that dopaminergic modulation of the BLA originates not only from the LC but also from canonical dopamine-producing brain regions such as the ventral tegmental area (VTA). To disambiguate this dual-source contribution, we performed complementary experiments and the results revealed robust activation of LC^TH^ neurons during restraint stress, whereas VTA^TH^ neurons showed no such activation (Supplementary Fig. 14). However, the specific subpopulations of VTA^TH^ neurons that may be involved in the PTSD relapse model used in this study remain to be elucidated.

There are several limitations in this study. First, activity-tagging technique was used to selectively label memory engram cells in BLA. Although we have clarified that BLA^engram^ has a crucial role in relapse of extinguished fear, the molecularly distinct cell type of BLA engram cells is largely unknown. A recent study showed that fear extinction memory engram cells are encoded and stored in the Ppp1r1b-positive (Ppp1r1b^+^) subpopulations of BLA neurons and that these engram cells are essential for suppressing the fear memory trace^55^. Further research should be undertaken to investigate the role of BLA Ppp1r1b^+^neuronal populations in relapse of extinguished fear and PTSD. Second, the effect of norepinephrine was released by LC^TH^ neurons in Ext-Res intervention for fear relapse. We infused dopamine into the BLA after extinction to mimic the therapeutic effect and pharmacologically blocked D1 or D2 receptors in the BLA via intracranial injection of antagonists. These results are consistent with those of Sofia Beas (2018) who discovered that LC drives disinhibition in the paraventricular nucleus of the thalamus via a dopamine D2 receptor mechanism^22^, and Kimberly (2016) found that dopamine release from the LC into the dorsal hippocampus enhanced spatial learning and memory^19^. However, the necessity and sufficiency of BLA norepinephrine levels and its receptors are still little known. Further work is required to clarify the function of norepinephrine and its receptors in the process of fear memory trace erased by Ext-Res intervention.

Taken together, these findings demonstrate that restraint intervention activates LC^TH^ neurons and then inhibits the BLA^engram^ activation via a dopamine D2-dependent mechanism, thereby erasing fear memory trace to prevent relapse of extinguished fear. Our results have broad implications for our understanding of the circuit mechanisms of relapse of extinguished fear and suggest that further study of the dopaminergic circuit from LC^TH^ neurons to BLA^engram^ and dopamine signaling pathways should greatly advance our current understanding of PTSD relapse after exposure therapy.

## Supplementary information

## Data Availability

The data that support the findings of this study are available from the corresponding author upon reasonable request.
