# Supplementary material for: Extinction-restraint intervention eradicates fear memory encoded in BLA engram cells via D2 receptor-mediated dopaminergic modulation from locus coeruleus
Source: Exp Mol Med. 2026 Aug 13;58(8):2762–80. doi: 10.1038/s12276-026-01794-0 (PMC13538692; doi:10.1038/s12276-026-01794-0)
Supplement: Supplementary file 1 [file 12276_2026_1794_MOESM1_ESM.pdf]

Supplementary information

Extinction-restraint intervention eradicates fear memory encoded in BLA engram cells via D2 receptor-mediated dopaminergic modulation from locus coeruleus

Authors

Tongxia Li [1] †, Jian Yang [2]†, Chi Cui [1], Kun Ren [2], Lijun Zhang [2], Ming Li [2], Xueke Yang [2], Xiang Peng [2], Jie Lei [2], Yulong Shi [2], Gangan Luo [2], Yibo Yao [2], Junsong Du [2], Sitong Chen [2], Jie Ming [3], Xuemin Wang [4*], Pei Zhang [2*], Bo Tian [1,5,6*]

Affiliations

1 *Hubei Provincial Clinical Research Center for Alzheimer's Disease, Tianyou Hospital, School of Medicine, Wuhan University of Science and Technology, Wuhan, Hubei, 430081, China; Department of Neurology, Tongji Hospital, Tongji Medical College, Huazhong University of Science and Technology, Wuhan, Hubei, 430030, China.*

2 *Department of Neurobiology, School of Basic Medicine, Tongji Medical College, Huazhong University of Science and Technology, Wuhan, Hubei Province, 430022, P. R. China.*

3 *Department of Breast and Thyroid Surgery, Union Hospital, Huazhong University of Science and Technology, Wuhan, Hubei Province, 430022, P. R. China.*

4 *Department of Neurobiology, Southern Medical University, Guangzhou, Guangdong Province, 510515, P. R. China.*

5 *Hubei Province Key Laboratory of Occupational Hazard Identification and Control, Wuhan University of Science and Technology, Wuhan, Hubei, 430081, China.*

6 *Academy of Advanced Interdisciplinary Research, Wuhan University of Science and Technology, Wuhan, Hubei, 430081, China.*

†*These authors contributed equally to this work*

[*] *Correspondence authors*

Corresponding authors at: School of Medicine, Wuhan University of Science and Technology, Wuhan, Hubei Province, 430081, P.R. China

E-mail address: xmwang@smu.edu.cn (X. Wang), zhangpei@hust.edu.cn (P. Zhang), tianbo@wust.edu.cn(B. Tian)

Lead contact: tianbo@wust.edu.cn(B. Tian)

Supplemental Figures

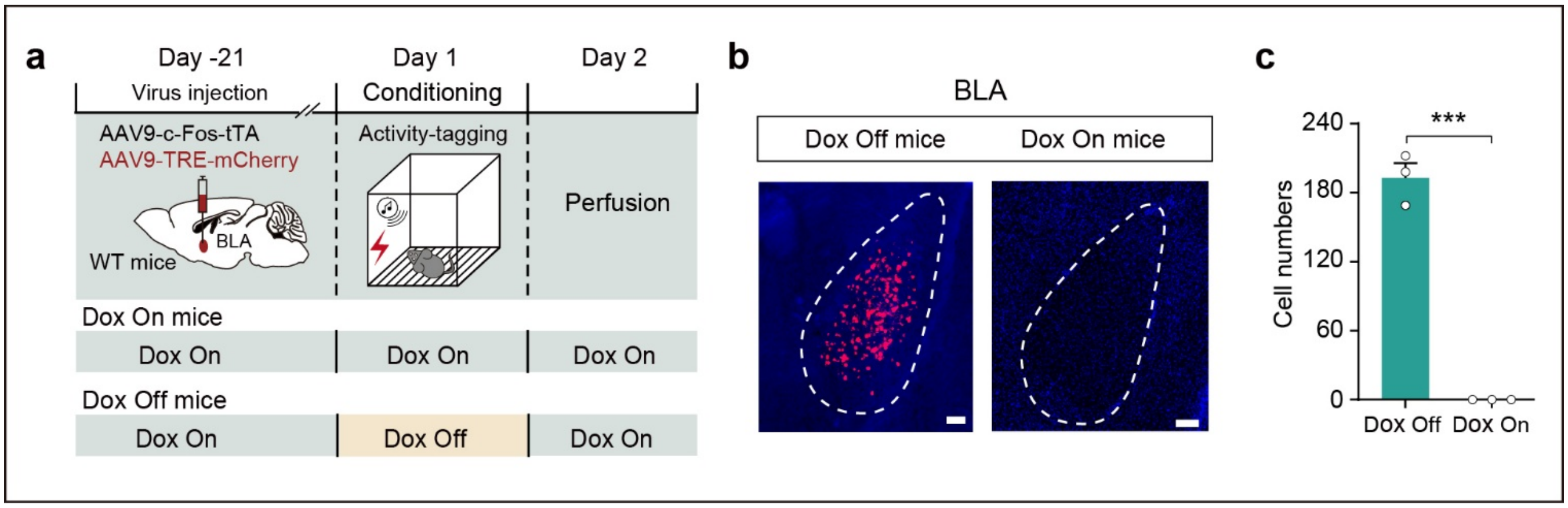

Supplementary Fig. 1. Validation of c-fos-tTA/TRE Tet-off system.

a Schematic of viral injections and Dox diet control. IF, immunofluorescence. **b** Representative image of mCherry-expressing cells in BLA. Blue for DAPI. (Scale bar, 100 μm). **c** Statistical analysis of mCherry^{+} cells in BLA (n = 3 mice per group). Data are presented as mean ± SEM. ***P < 0.001.

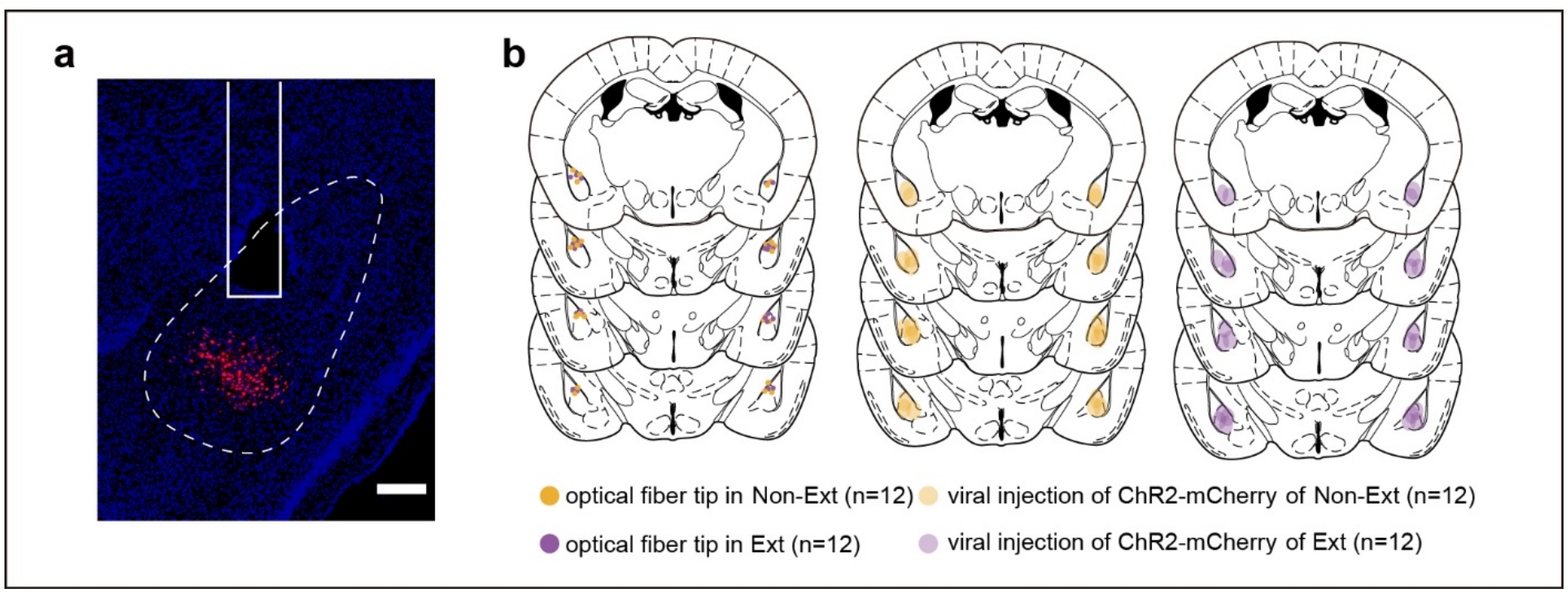

Supplementary Fig. 2 Representative images and fiber placement diagrams Related to Fig. 2a-e.

a Representative image of ChR2 expression in BLA site for Fig. 2**a-e**. (Scale bar, 200 μm). **b** Bilateral optic-fiber tip positions and viral injection coordinates for every animal represented in Fig. 2**b-e.**

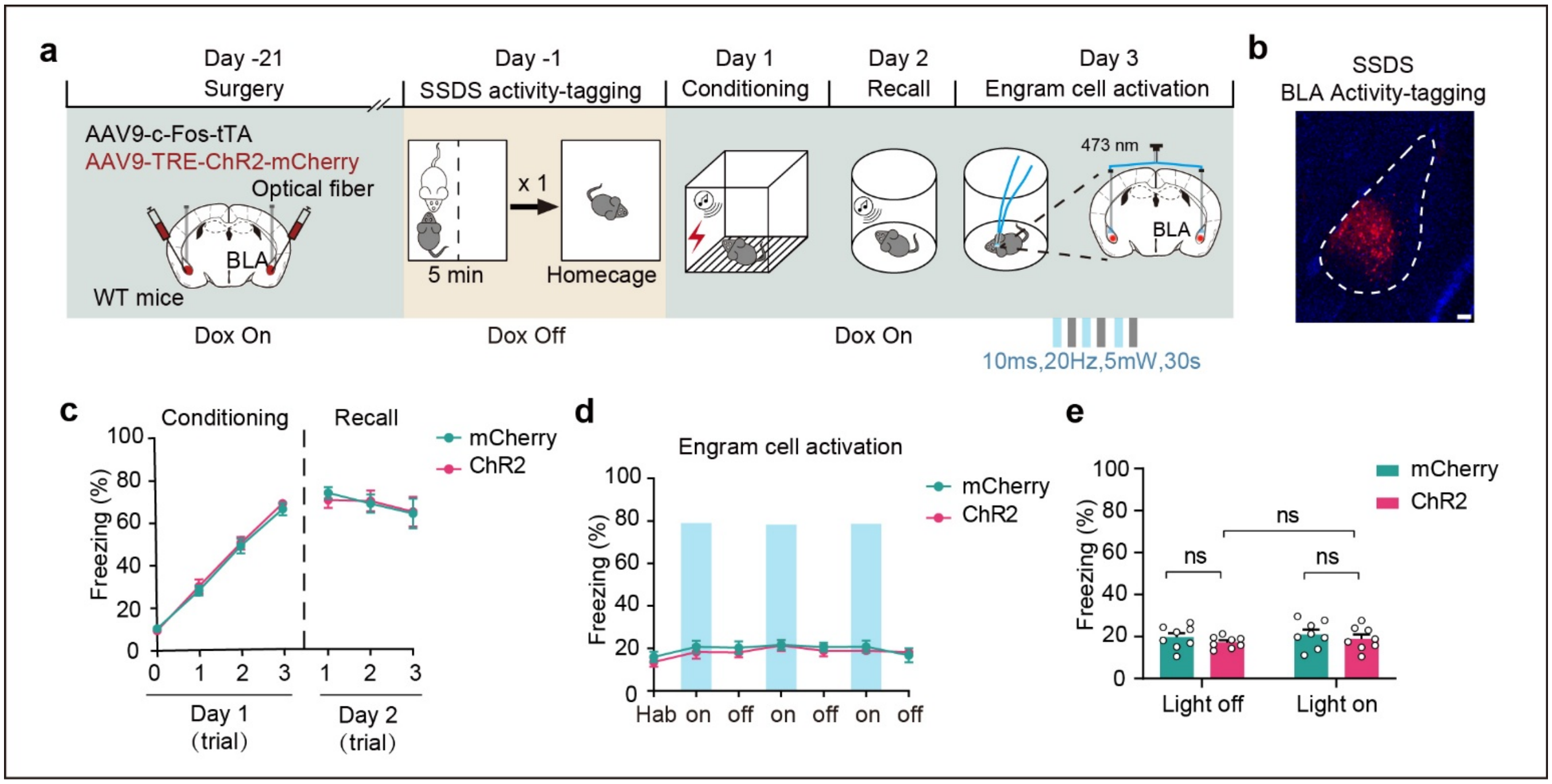

Supplementary Fig. 3 Activation of single social defeated stress-related engram cells in BLA does not induce freezing behavior.

a Schematic of viral strategy and mouse behavior tests. Single social defeated stress, SSDS. **b** Representative fluorescence image of BLA mCherry-expressing cells via the Dox Off activity-tagging (Scale bar, 100 μm). **c-e** Statistical results of freezing levels in different phases (n = 8 mice per group). Data are presented as mean ± SEM. ns for no significance.

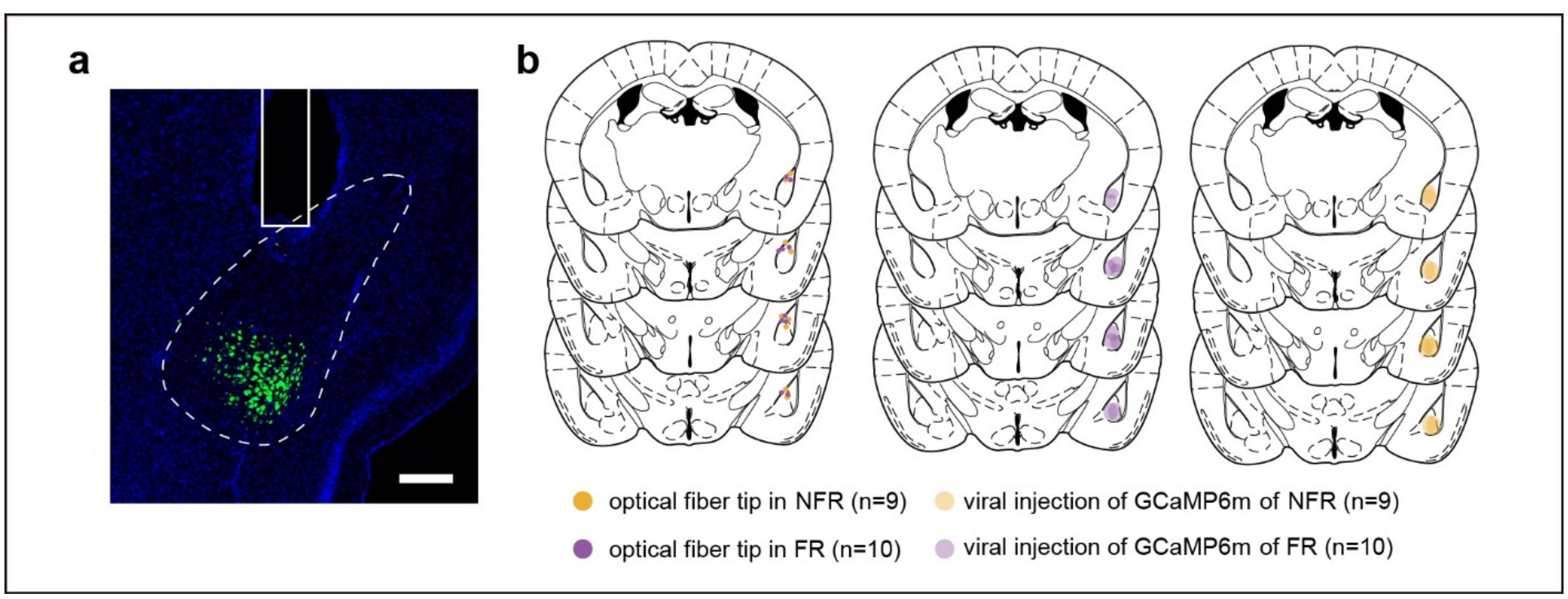

Supplementary Fig. 4 Representative images and fiber placement diagrams Related to Fig. 2g-m.

a Representative image of GCaMP6m expression in BLA for Fig. 2**g-m**. (Scale bar, 200 μm). **b** Optic-fiber tip locations and viral injection sites for all animals included in Fig. 2**h-m**.

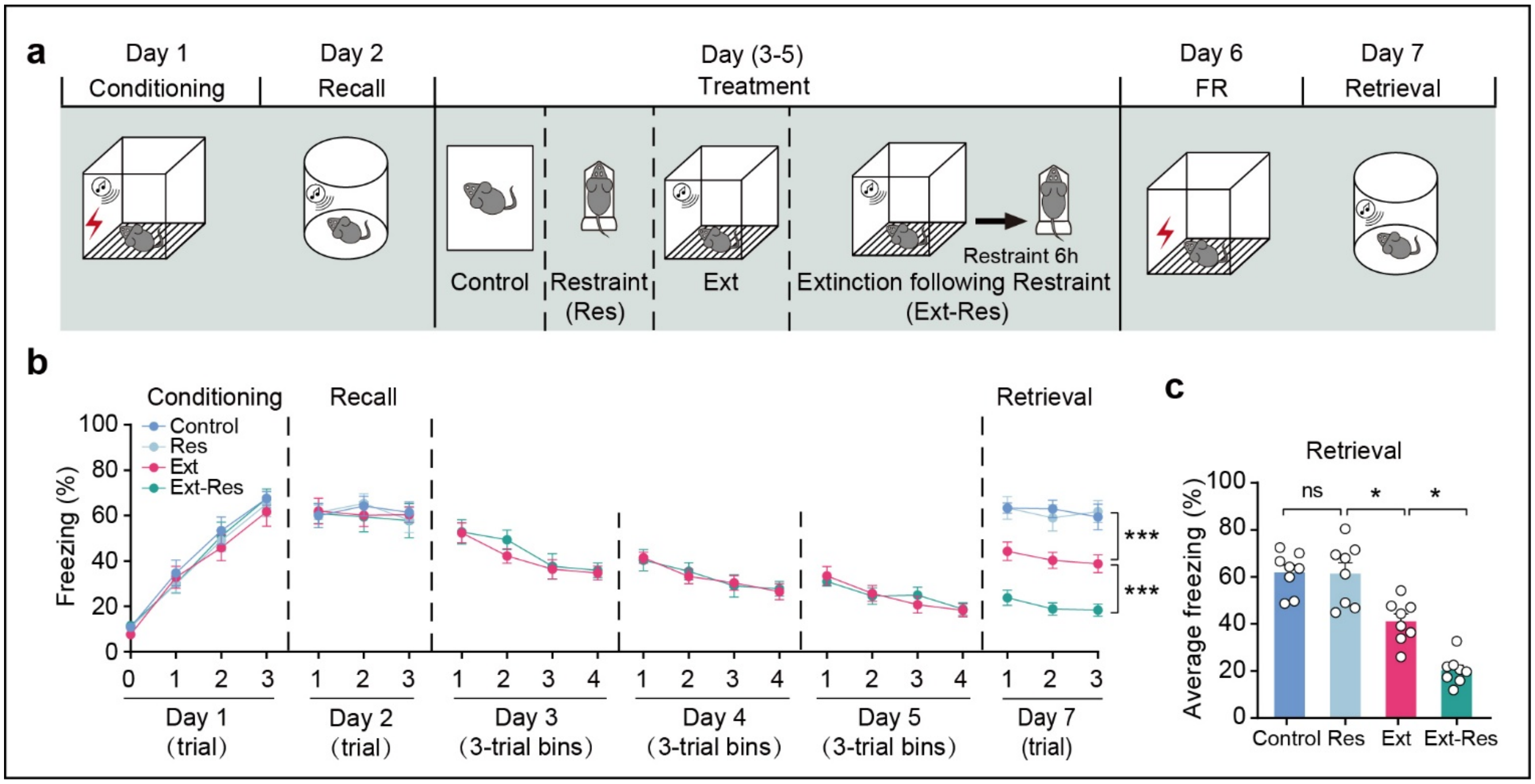

Supplementary Fig. 5 Restraint with extinction prevents fear relapse, but restraint or extinction alone does not.

a Experimental timeline of fear conditioning, interventions, and behavior tests. **b** Freezing levels across conditioning, recall, extinction, and retrieval phases in four groups. **c** Statistical analysis of freezing levels during the retrieval phase. Data are showed as mean ± SEM. **P* < 0.05, ****P* < 0.001, and ns for no significance.

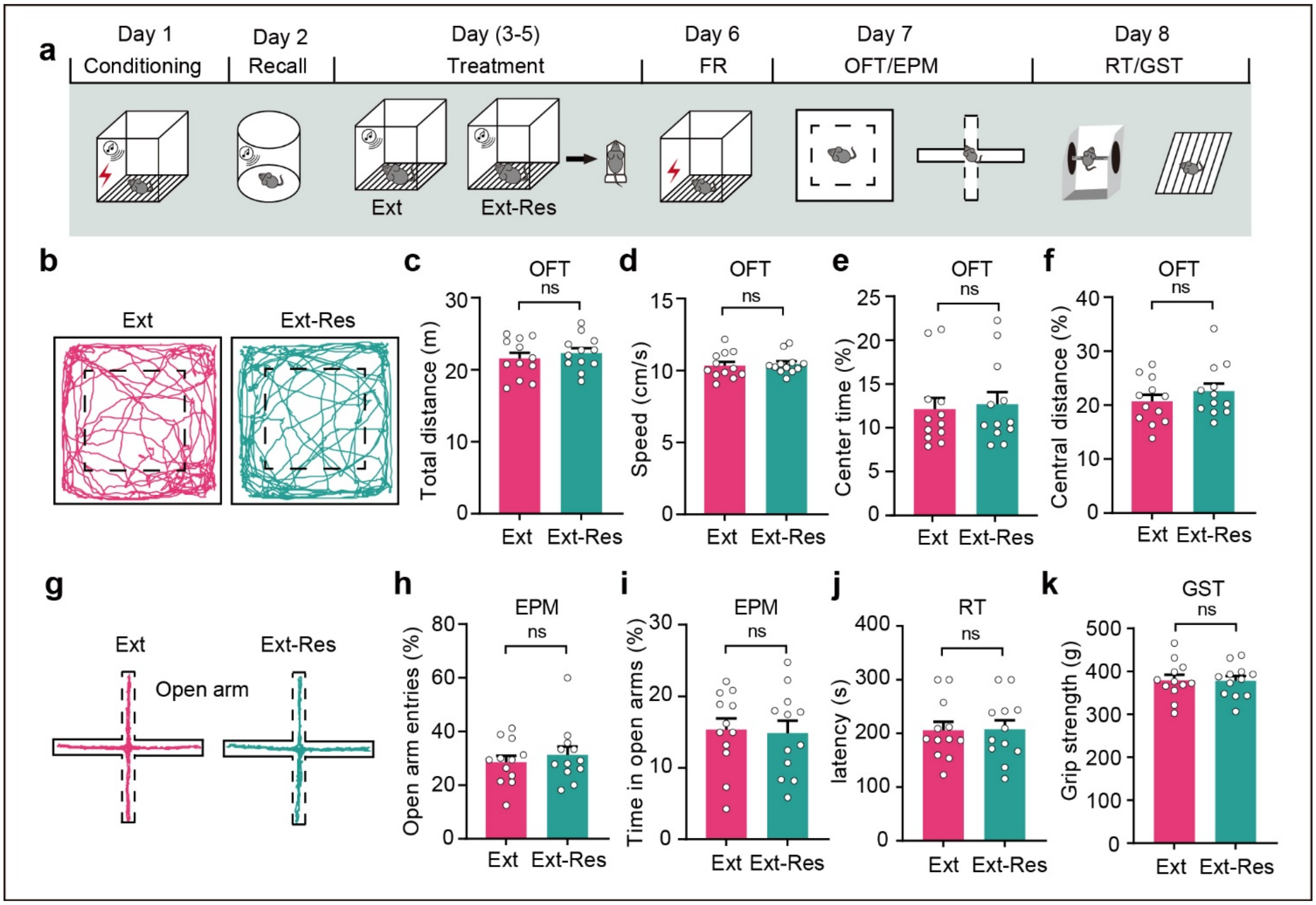

Supplementary Fig. 6 Restraint with extinction prevents fear relapse, but restraint or extinction alone does not.

a Timeline of the experiments performed. **b-f** Traces and statistics in open field test. **g-i** Traces and statistics in elevated plus maze. **j-k** Rotarod test (**j**) and grip strength test. Open field test, OFT. Elevated plus maze, EPM. Rotarod test, RT. Grip strength test, GST. Data are showed as mean ± SEM. *P < 0.05, ***P < 0.001, and ns for no significance.

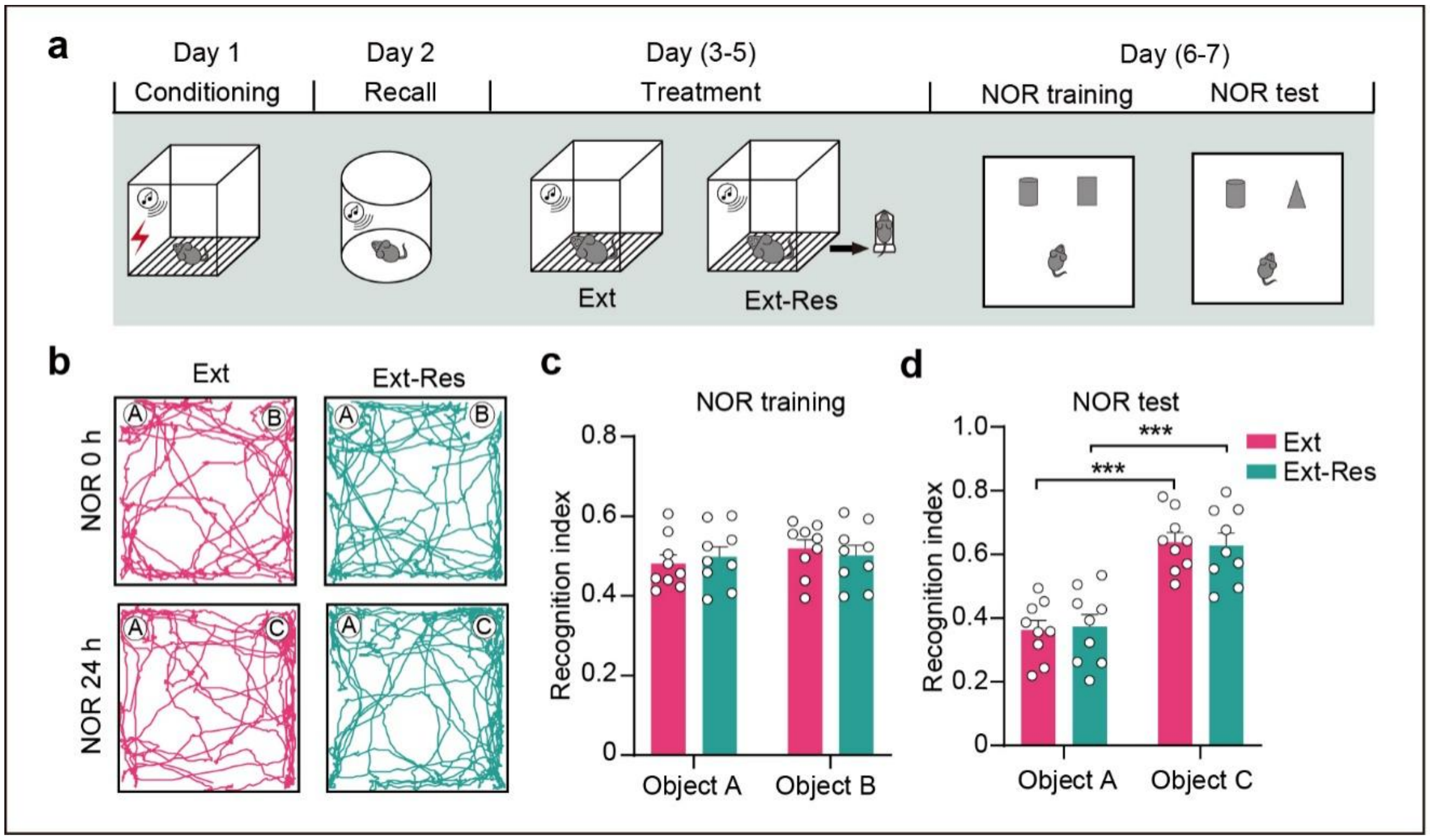

Supplementary Fig. 7 Restraint has no discernible impact on cognitive performance.
a Schematic diagram of the experimental design. **b** Representative trajectories during the NOR testing**.** **c-d** The recognition index to explore object on NOR training (**c**) and testing (**d**). Data are presented as mean ± SEM. ***P < 0.001.

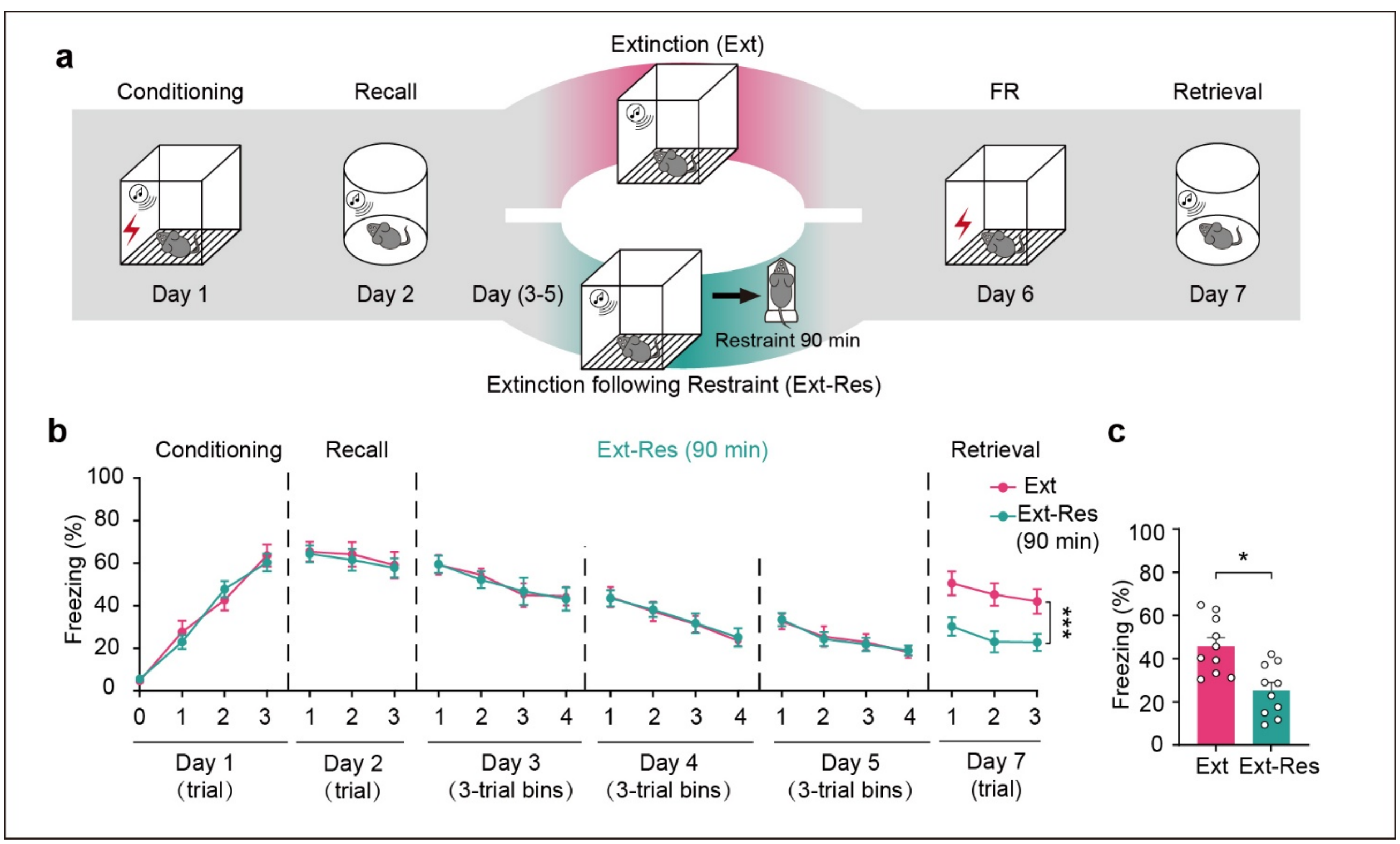

Supplementary Fig. 8 Restraint intervention with 90-min can alleviate fear relapse.

a Schematic diagram of the experimental design. **b** Behaviour results of restraint 90-min restraint intervention. **c** Freezing levels during the retrieval phase. Data are presented as mean ± SEM. **P* < 0.05, ****P* < 0.001.

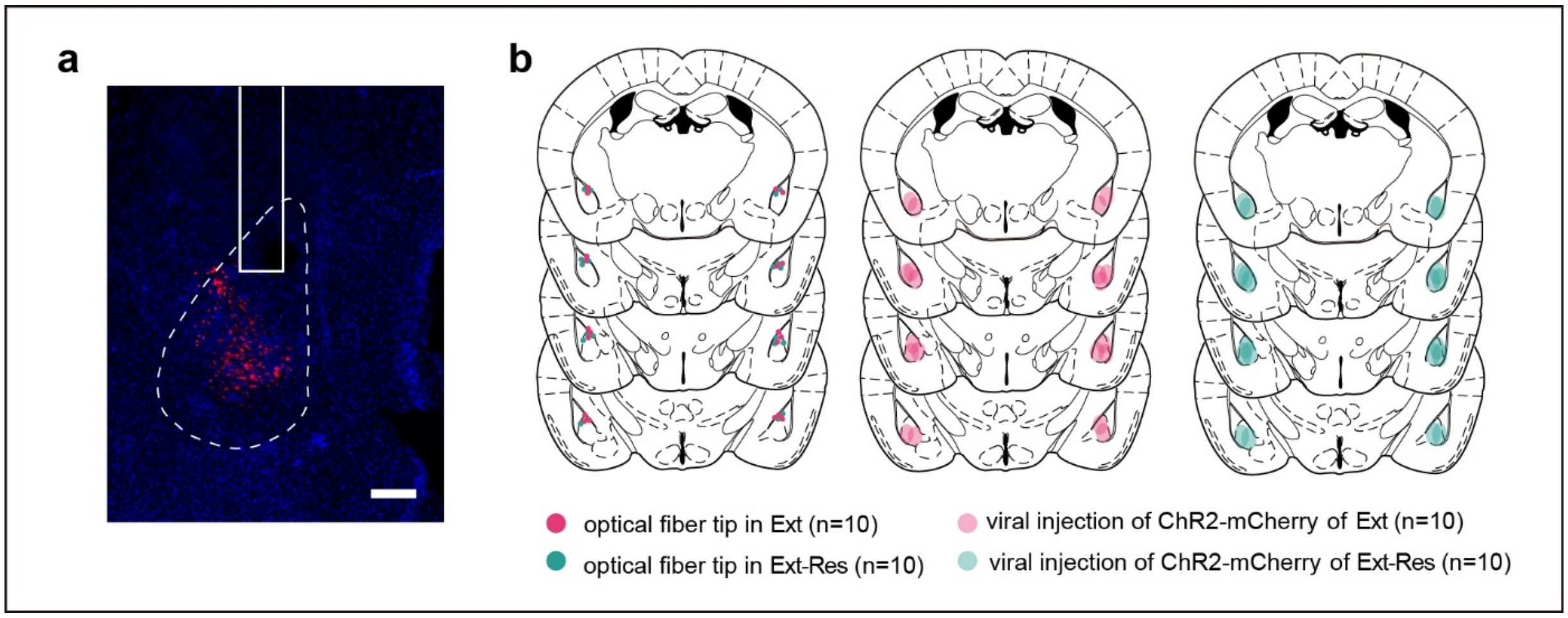

Supplementary Fig. 9 Representative images and fiber placement diagrams Related to Fig. 4a-e.

a Representative image of ChR2 expression in BLA for Fig. 4**a-e** (Scale bar, 200 μm). **b** Bilateral optic-fiber tip positions and viral injection coordinates for every animal represented in Fig. 4**b-e**

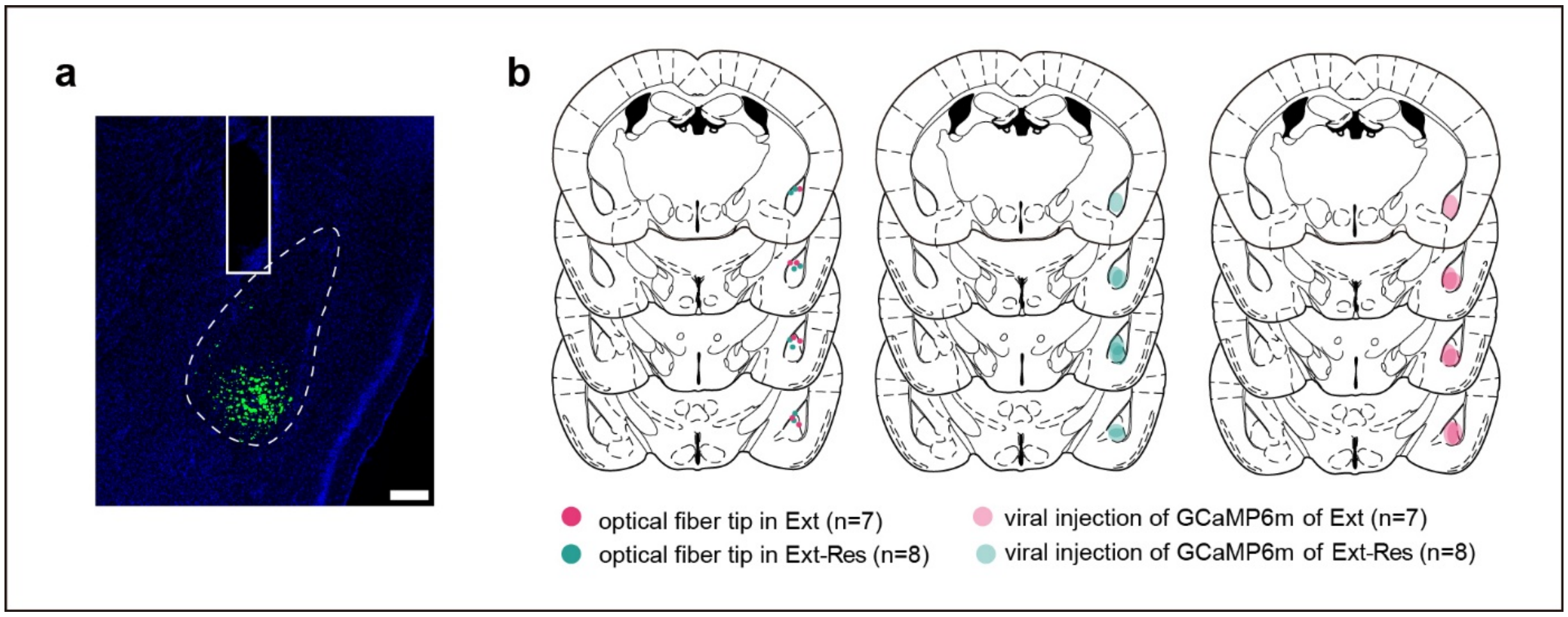

Supplementary Fig. 10 Representative images and fiber placement diagrams Related to Fig. 4f-l.

a Representative image of GCaMP6m expression BLA for Fig. 4**f-l** (Scale bar, 200 μm)**. b** Optic-fiber tip locations and viral injection sites for all animals included in Fig. 4**g-l**.

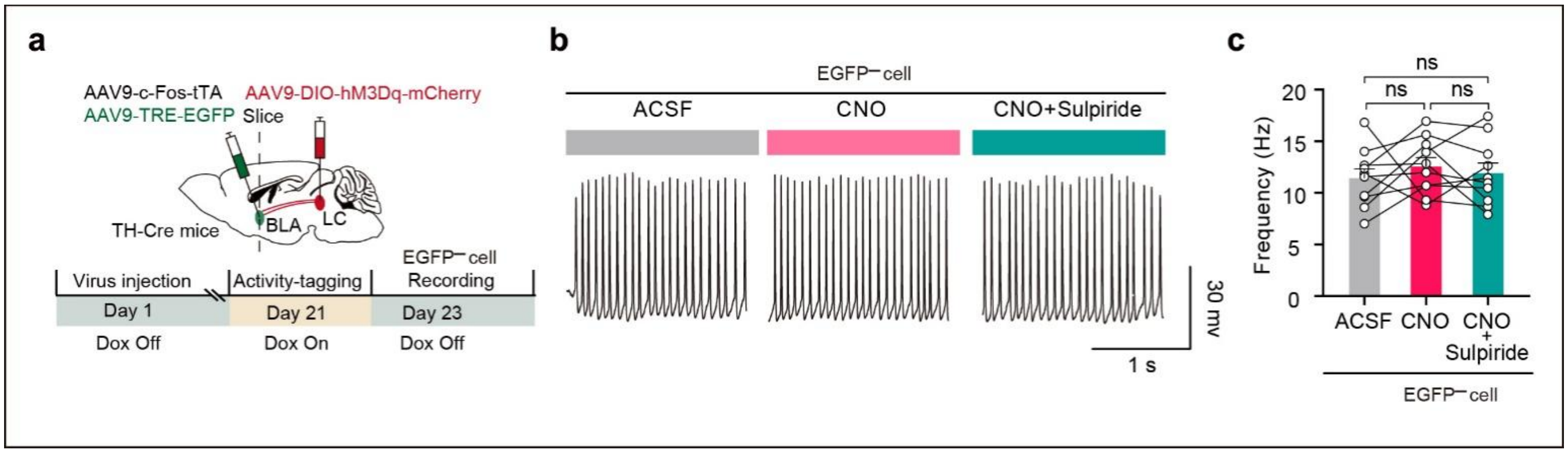

Supplementary Fig. 11 LC terminal activation or D2 receptor antagonism does not affect firing rates of non-tagged BLA neurons.

a Experimental program for electrophysiology recordings in vitro. **b-c** Representative traces with ACSF, CNO, and CNO+Sulpiride treatment in non-tagged BLA neurons (EGFP⁻ cell). Statistical results of non-tagged BLA neurons firing rates after chemogenetic activation of LC TH⁺ terminals in different conditions (**c**). Data are showed as mean ± SEM. ns for no significance.

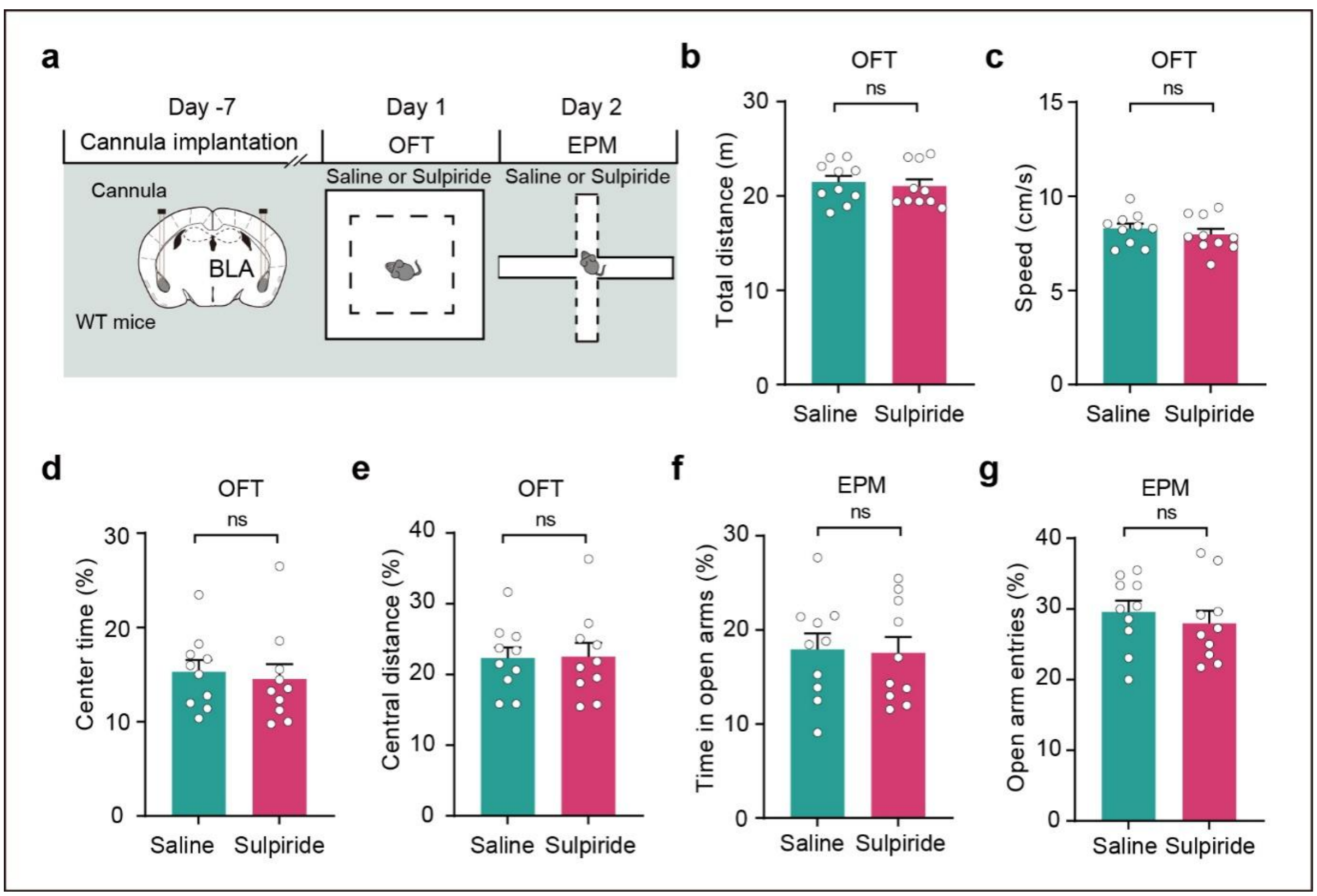

Supplementary Fig. 12 Intracranial injection of D2R antagonist in BLA does not affect locomotor activity and anxiety-like behavior.

a Schematic of behavioral tests. **b-e** Total distance, speed, center time, and central distance in open field test (OFT). **f-g** Statistical results in elevated plus maze (EPM) test.

Data are showed as mean ± SEM. ns for no significance.

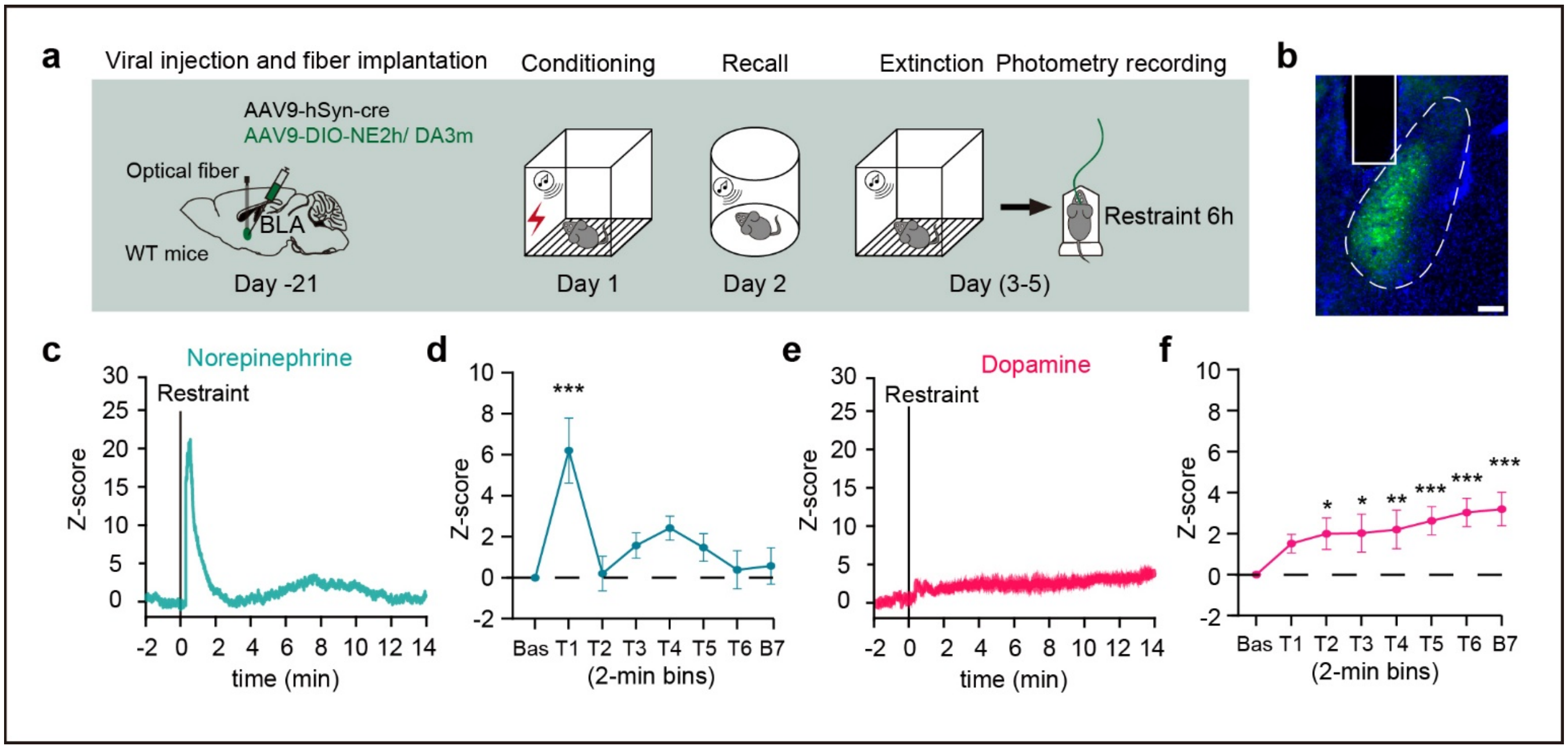

Supplementary Fig. 13 Bulk BLA NE2h/ DA3m Fiber Photometry During Restraint.

a Experimental protocol for fiber-photometry recording of bulk BLA neuronal NE2h and DA3m sensor signals during restraint treatment. **b** Representative images of viral expression in the BLA. (Scale bar, 200 μm). **c** Traces of norepinephrine fluorescence dynamics during the session before restraint and after restraint treatment. **d** Mean response for norepinephrine fluorescence signals z-score for 2 min-bins during restraint. **e** Traces of dopamine fluorescence dynamics during the session before restraint and after restraint treatment. **f** Mean response for dopamine fluorescence signals z-score for 2min-bins during restraint. Data are showed as mean ± SEM. *P < 0.05, **P < 0.01, ***P < 0.001.

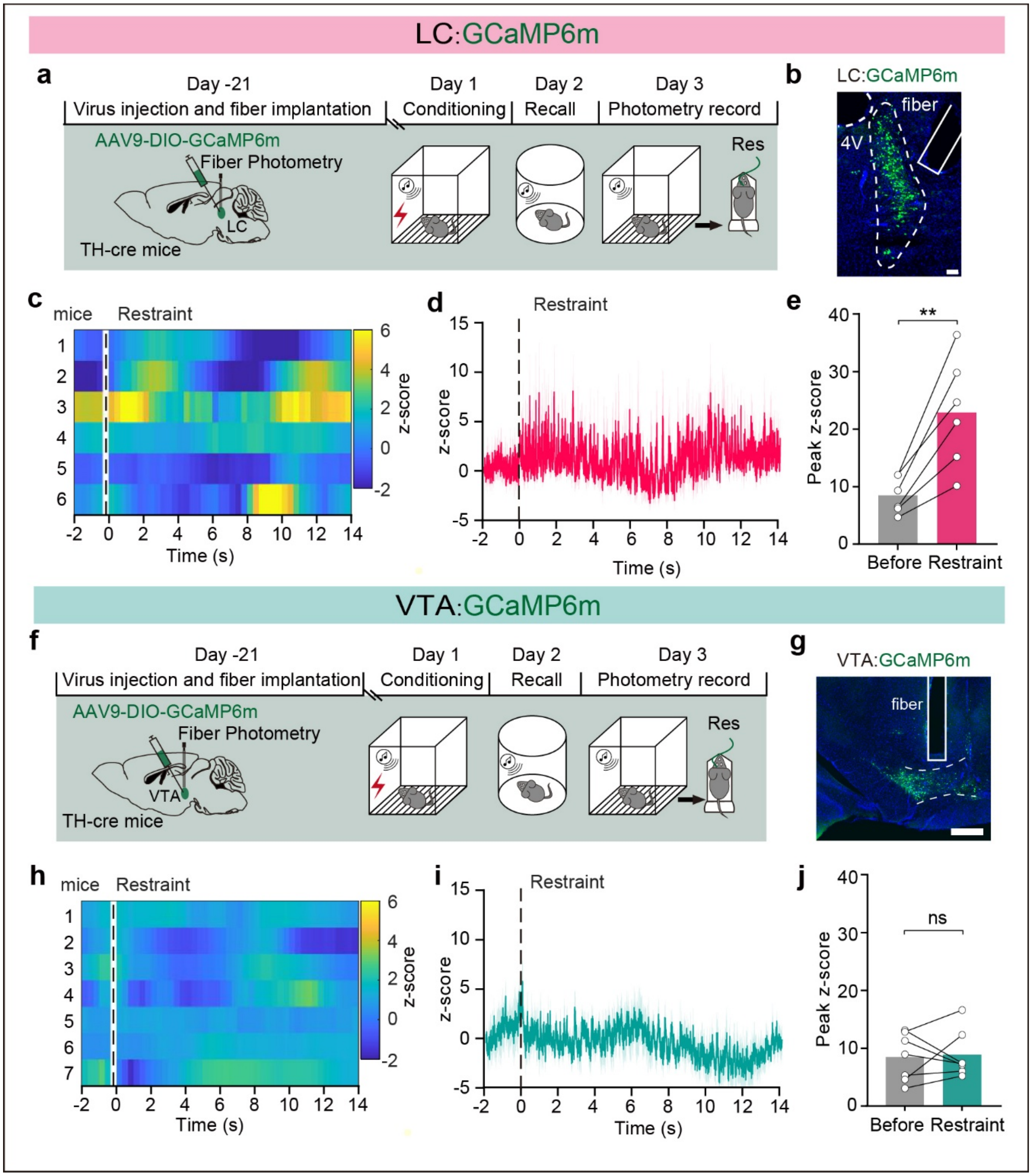

Supplementary Fig. 14 Restraint treatment significantly activates LC^{TH} neurons, but not midbrain dopamine neurons.

a Experimental timeline for viral strategy and fiber photometry. **b** Representative image of viral expression and optical fiber site in LC. (Scale bar, 100 μm). **c** Heatmap visualizing the changes of LC^{TH} fluorescence signals (z-score) in response to restraint (n = 6 mice). **d** Mean response of LC^{TH} neurons during Ext-Res. **e** Peak fluorescence changes before and after restraint. **f-j** Similar to (**a-e**), but analysis for VTA. Data are displayed as mean ± SEM. $^{**}P < 0.01$, and ns for no significance.
